# A Pan-Cancer Analysis of Tumor-Infiltrating B Cell Repertoires

**DOI:** 10.3389/fimmu.2021.790119

**Published:** 2022-01-05

**Authors:** Katharine Yu, Akshay Ravoor, Núria Malats, Silvia Pineda, Marina Sirota

**Affiliations:** ^1^ Bakar Computational Health Sciences Institute, University of California, San Francisco (UCSF), San Francisco, CA, United States; ^2^ Department of Pediatrics, University of California, San Francisco (UCSF), San Francisco, CA, United States; ^3^ Genetic and Molecular Epidemiology Group, Spanish National Cancer Research Centre (CNIO), and Centro de Investigación Biomédica en Red Cáncer (CIBERONC), Madrid, Spain

**Keywords:** B cell repertoire, immune repertoire, tumor microenvironment, tumor infiltration, TCGA

## Abstract

Tumor-infiltrating B cells can play an important role in anti-tumor responses but their presence is not well understood. In this study, we extracted the B cell receptor repertoires from 9522 tumor and adjacent non-tumor samples across 28 tumor types in the Cancer Genome Atlas project and performed diversity and network analysis. We identified differences in diversity and network statistics across tumor types and subtypes and observed a trend towards increased clonality in primary tumors compared to adjacent non-tumor tissues. We also found significant associations between the repertoire features and mutation load, tumor stage, and age. Our V-gene usage analysis identified similar V-gene usage patterns in colorectal and endometrial cancers. Lastly, we evaluated the prognostic value of the repertoire features and identified significant associations with survival in seven tumor types. This study warrants further research into better understanding the role of tumor-infiltrating B cells across a wide range of tumor types.

## 1 Introduction

While B cells are well-established as an integral part of the adaptive immune system, only recently studies began to elucidate their role in cancer (1, 2). The number of studies on tumor-infiltrating B cells is vastly eclipsed by the number of studies on tumor-infiltrating T cells, the latter of which have largely been the focus of researchers and play a central role in modern immunotherapies such as checkpoint inhibitors. However, B cells hold great potential for the development of new immunotherapies and as biomarkers for immunotherapy response.

A main function of B cells is to recognize specific antigens with the immunoglobulins (Ig), or B cell receptors (BCR), on their cell surface. These Ig are made up of two heavy chains (IGH) and two light chains, the kappa (κ) chains (IGK) or the lambda (λ) chains (IGL). Ig are generated through a process called somatic recombination where variable (V), diversity (D), and joining (J) gene segments are randomly combined to create a diverse collection of antigen receptors which can recognize a wide range of antigens. Additionally, B cells undergo a process called somatic hypermutation (SHM) upon antigen binding which introduces additional mutations into the variable regions of the Ig genes, further diversifying the receptors.

The collection of diverse B cell receptors within an individual, or the B cell repertoire, can be interrogated using high-throughput technologies. B-cell receptor sequencing (BCR-seq) is commonly used to study the B cell repertoire as it offers greater sensitivity compared to unselected RNA-seq by targeting the BCR region rather than the entire transcriptome. However, the amount of publicly available RNA-seq datasets is much greater than the number of BCR-seq datasets. Tools such as MiXCR (3), ImReP (4), and TRUST4 (5) have been developed to extract BCR reads from bulk RNA-seq data and align them to the V, D, and J gene segments, allowing for the characterization of the immune repertoire from sequencing data. These tools have been especially useful in mining publicly available datasets to extract insight into the adaptive immune system (6, 7).

The Cancer Genome Atlas (TCGA) is the largest publicly available dataset of molecularly characterized human tumors (8). The data generated by the TCGA includes clinical, transcriptomic, methylation, mutation, copy number, and proteomics data. This dataset has greatly advanced our understanding of tumor biology and has led to improvements in cancer diagnosis, treatment, and prevention (9–12). More recently, studies have leveraged the TCGA dataset to investigate the role of the immune system in cancer (7). However, the previous analysis of the B cell repertoires tends to be limited in scope and lacks tumor subtype stratification. For example, Thorsson et al. only analyzed the impact of TCR diversity on prognostic associations and their BCR analysis was restricted to comparing BCR diversity across tumor types and the immune subtypes identified in the paper.

Characterization of the tumor microenvironment is vital for understanding cancer biology and developing new immunotherapies as well as predicting which patients will respond to immunotherapies. B cells, in particular, can play an important role in the antitumor immune response. They can produce antibodies which can drive antibody-dependent cellular cytotoxicity and phagocytosis of tumor cells (13) and they can also present antigens to T cells and may be involved in the formation of tumor-associated tertiary lymphoid structures (14, 15). However, the presence of tumor-infiltrating B cells has also been associated with poor outcome in renal cell carcinoma (16), bladder cancer (17), prostate cancer (18), suggesting that B cells play a complex role in the tumor microenvironment. Further studies are needed to better understand how tumor-infiltrating B cells behave in different tumor contexts.

We extracted the B cell repertoires from 28 tumor types in the TCGA dataset for nearly 10,000 samples and performed diversity and network analysis to investigate the immunological differences and commonalities across tumor types and subtypes, and between tumors and adjacent non-tumor tissue. We then compared these B cell repertoire features to host, clinical, and molecular features and found significant associations with age, tumor stage, and mutation load, respectively. In our V-gene analysis, we found similar V-gene usage patterns in colorectal and endometrial cancers. Lastly, we investigated the prognostic value of each repertoire feature and found significant associations with survival in a subset of tumor types.

## 2 Methods

### 2.1 Data Acquisition

We used the GDC Data Transfer Tool (https://gdc.cancer.gov/access-data/gdc-data-transfer-tool) to download every available TCGA RNA-Seq FASTQ file from the GDC Legacy Archive (https://portal.gdc.cancer.gov/legacy-archive/search/f). We then used MiXCR to extract the reads that align to the VDJ region of the IGH, IGK, IGL, TRA, TRB, TRD, and TRG chains using the MiXCR pipeline for processing RNA-seq and non-targeted genomic data (https://mixcr.readthedocs.io/en/master/rnaseq.html). We filtered out reads with missing CDR3 sequences. We used the R package GenomicDataCommons (19) to annotate the samples with their TCGA barcodes and extracted the sample type from the TCGA barcode. We then filtered for primary tumor samples and adjacent non-tumor samples for all the tumor types except for SKCM, where we included metastatic samples as well. If there were multiple vials from the same tumor sample, we selected the first vial (e.g. -01A).

We downloaded the TCGA clinical data from the TCGA Pan-Cancer Atlas Hub hosted by the UCSC Xena platform (https://pancanatlas.xenahubs.net). We downloaded the leukocyte fraction data and mutation load data from the PanCanAtlas Publications page on GDC for The Immune Landscape of Cancer (https://gdc.cancer.gov/about-data/publications/panimmune). We used the R package TCGAbiolinks (20) to download the TCGA subtype data and we used the “Subtype_Selected” column for the subtype information if there were multiple subtype classifications.

### 2.2 Expression and Diversity Analysis

We calculated BCR expression by dividing the number of reads that align to each IGH, IGK, or IGL chain by the total number of reads in each sample. We defined clones as groups of reads that share the same V and J genes, the same CDR3 length, and at least 90% shared nucleotide identity. As it is not possible to perform paired heavy and light chain analysis with bulk RNA-seq data, we analyzed the IGH, IGK, and IGL chains separately for the clonal analysis. To quantify the clonal diversity, we calculated Shannon entropy (H) using the following formula:


$$H=-\sum_{i=1}^{N}p_{i}\log_{2}p_{i}$$

N is the number of unique clones in the sample and p_i_ is the proportion of clone *i* in the sample. Shannon entropy can range from 0, for samples with only one clone, to log_2_N, for samples with a uniform distribution of clones. We then calculated the evenness of each sample using Pielou’s evenness index, which is:


$$J=\frac{H}{H_{\max}}$$

where *H* is the Shannon entropy and *H_max_* is the maximum possible value for *H*. Evenness is constrained between 0 and 1 and a higher evenness value indicates a more even distribution of clones.

### 2.3 Network Analysis

We generated networks for each sample using a previously published method (21, 22). Each vertex in the network is a unique BCR sequence and the size of the vertex corresponds to the number of reads with that sequence. Edges are drawn between vertexes with the same V and J genes, the same CDR3 length, and at least 90% sequence similarity (our clone definition). We used the R package igraph to generate network plots for each sample.

We used the Gini index to quantify different repertoire network parameters. The Gini index measures the inequality in a frequency distribution and it ranges from 0, which indicates complete equality, to 1, which indicates complete inequality. We quantified clonal expansion by calculating the Gini index using the distribution of vertex sizes for each sample (vertex Gini index). This measures the unevenness in the number of unique BCR sequences and having a higher vertex Gini index indicates more clonal expansion in a sample. We quantified clonal diversification by calculating the Gini index using the distribution of the number of vertexes in each cluster (cluster Gini index). Having a higher cluster Gini index indicates a sample with expanded cluster sizes.

For the downsampling analysis, we randomly sampled 500 reads each for IGH, IGK, and IGL and then calculated the vertex Gini index and the cluster Gini index using these subsamples. We repeated this procedure 10 times and then took the mean for the final analysis presented in the paper.

### 2.4 V-Gene Analysis

To analyze V-gene usage, we first filtered out samples with fewer than 100 clones. We then calculated V-gene usage as the percent of clones in each sample which uses a particular V-gene. We used PCA to reduce the dimensionality of the data and we visualized the samples in PC1 and PC2 to identify clusters of interest. Next, we applied an elastic net model to identify the genes that are associated with these clusters of interest. We used 5-fold cross validation to test different alpha values (0.1-0.9) and selected the alpha value with the lowest mean cross validated error for the final elastic net model.

### 2.5 Statistical Analysis

We used R version 4.3 to perform the statistical analysis and generate the figures in this paper. For the association analysis between B cell repertoire features and clinical and tumor features, we used Spearman’s correlation for continuous variables and the Wilcoxon rank-sum test for categorical variables. We used the R package survival to perform the Cox regression analysis and we used the R package survminer to generate the Kaplan-Meier plots. For analysis involving multiple B cell repertoire features for each tumor type, we adjusted for multiple comparisons within tumor types using the Benjamini-Hochberg procedure.

## 3 Results

### 3.1 Study Overview

**Figure 1:**
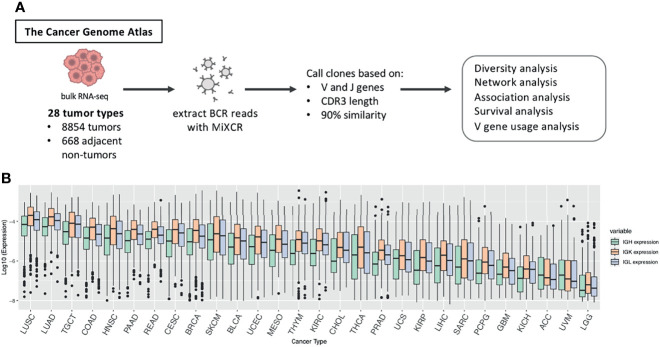
Study overview. **(A)** BCR reads were extracted from TCGA RNA-seq data across 28 tumor types using MiXCR and we called clones based on sequences having the same V and J gene, the same CDR3 length, and at least 90% sequence similarity. We then performed diversity analysis, network analysis, association analysis, survival analysis, and V gene usage analysis to investigate differences in the B cell immune repertoire across tumor types and between tumor and adjacent non-tumor samples. **(B)** Boxplots showing the log10 expression of IGH, IGK, and IGL. Expression is defined as the number of reads for each chain divided by the total number of reads in the sample. The box plot depicts the median as well as the upper and lower quartiles, and the whiskers depict 1.5 times the interquartile range.

We analyzed the B cell repertoires across the TCGA tumor samples corresponding to 28 tumor types with a total of 8854 tumor and 688 adjacent non-tumor samples (**Figure 1A** and **Supplementary Table 1**). We used MiXCR (3) to extract BCR sequences from RNA-seq data and align the sequences to the VDJ region of the IGH, IGK and IGL. As a quality control check, we verified that the number of immune repertoire reads extracted by MiXCR did not have a strong correlation with the total sequencing depth of each sample across tumor types (mean r = 0.148 (SD = 0.088) for tumor samples, mean r = 0.179 (SD = 0.232) for adjacent non-tumor samples, **Supplementary Figures S1A, B**). We defined expression of IGH, IGK, and IGL as the number of reads aligned to each chain divided by the total number of sequenced reads in each sample. **Figure 1B** and **Supplementary Table 1** shows the sequencing summary of BCR reads for all tumor types.

Many of the tumor types that have the highest IGH, IGK, and IGL expression such as lung squamous cell carcinoma (LUAD), lung adenocarcinoma (LUAD), head and neck squamous cell carcinoma (HNSC), and skin cutaneous melanoma (SKCM) (**Figure 1B**), are also the tumor types that have mutational burden as well as high leukocyte fractions (**Supplementary Figure 1C**), which was estimated by Thorsson et al. using methylation data (7), and are most responsive to checkpoint inhibitors (7). Likewise, the tumor types with the lowest expression of IGH, IGK, and IGL, such as uveal melanoma (UVM) and adrenocortical carcinoma (ACC), tend to have low leukocyte fractions and poor responses to immunotherapies (23, 24).

We also found that the expression derived from IGK are more abundant than IGH and IGL across nearly all of the tumor types (**Figure 1B**). This is similar to a previous study which analyzed Ig repertoires across 53 human tissues and found that CDR3 sequences account for 54% of the entire B-cell population on average (4).

### 3.2 Shannon Entropy and Evenness of BCR Repertoires Differ Across Tumor Types and Tend to Be Higher in Adjacent Non-Tumor Samples

**Figure 2:**
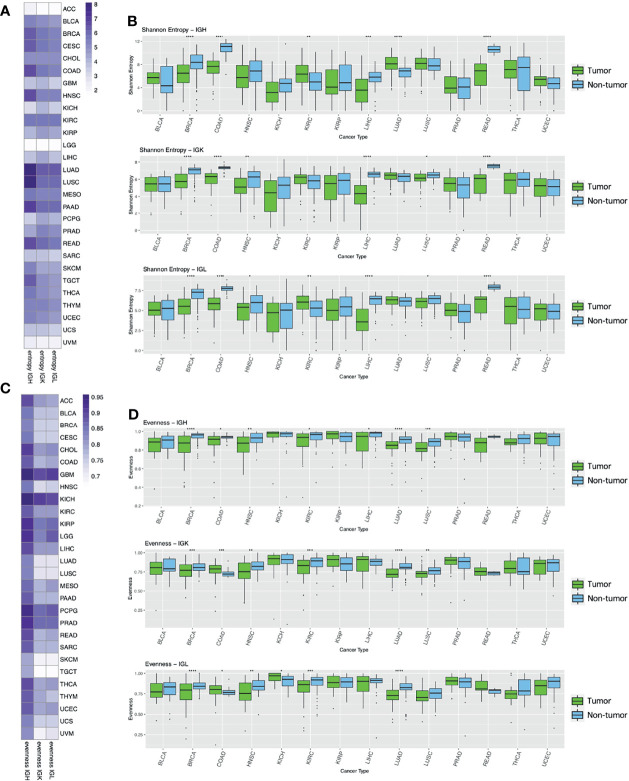
Entropy and evenness analysis across tumor types and between tumor and adjacent non-tumor samples. **(A)** The heatmap depicts the mean Shannon entropy value for each tumor type in each chain. **(B)** The boxplots show the Shannon entropy indexes for tumors (green) and adjacent non-tumor samples (blue) for the 14 tumor types with at least 10 adjacent non-tumor samples. Statistical significance was calculated using the Wilcoxon rank-sum test. Significant p-values are indicated by symbols above the box plots with one star corresponding to p-value < = 0.05, two stars corresponding to p-value < = 0.01, three stars corresponding to p-value < = 0.001, and four stars corresponding to p-value < = 0.0001. **(C)** The heatmap shows the mean Pielou’s evenness index for each tumor type in each chain. **(D)** The boxplots show the evenness index for tumors (green) and adjacent non-tumor samples (blue) and statistical significance was calculated as described above.

We defined clones as groups of reads that have the same V and J gene, the same CDR3 length, and at least 90% nucleotide similarity as in previous publications (21, 25). In order to quantify the diversity of Ig clones within each sample, we calculated the Shannon entropy within each Ig chain. Shannon entropy reflects both the number of clones as well as the frequency of the clones in each sample. We found that LUAD and LUSC have the highest Shannon entropy compared to the other tumor types (**Figure 2A**), which was unsurprising given the overall high Ig expression in these two tumor types. ACC, LGG, and UVM had the lowest Shannon entropy, which likely reflects the low expression of Ig in these tumor types. Overall, Shannon entropy was positively correlated with expression across all tumor types in the IGH, IGK, and IGL chains (**Supplementary Figure 2**). Interestingly, the correlation between Shannon entropy and expression was higher in IGH compared to IGK and IGL across tumor types. For example, the correlation (Spearman’s rho) between entropy and expression in LUAD is 0.69 for IGH but 0.27 and 0.35 for IGK and IGL, respectively. This suggests that there may be more uneven distributions of clones in IGK and IGL compared to IGH, which is reflected in IGK and IGL having lower Shannon entropy despite having higher expression than IGH.

We also observed that the tumor types that have higher diversity (LUAD, LUSC, BRCA, HNSC, KIRC, PAAD, READ, SKCM and TGCT) have a lower correlation with their expression values.

We then compared the Shannon entropy of primary tumor samples to adjacent non-tumor samples to better understand the tumor microenvironment. We analyzed the 14 tumor types (BLCA, BRCA, COAD, HNSC, KICH, KIRC, KIRP, LIHC, LUAD, LUSC, PAAD, READ, THCA, UCEC) that had at least 10 adjacent non-tumor samples and found that Shannon entropy was significantly higher in adjacent non-tumor samples compared to tumor samples in 4/14 (BRCA, COAD, LIHC, READ) tumor types for IGH, 6/14 (BRCA, COAD, HNSC, LIHC, LUSC, READ) tumor types for IGK, and 6/14 (BRCA, COAD, HNSC, LIHC, LUSC, READ) tumor types for IGL (**Figure 2B**). This trend could reflect a higher number of clones or a more even distribution of clones in the adjacent non-tumor samples compared to the tumor samples for these tumor types. Conversely, Shannon entropy was higher in tumor samples compared to adjacent non-tumor samples in only 2/14 (KIRC, LUAD) tumor types for IGH, none for IGK, and 1/14 for IGL (KIRC).

We then calculated Pielou’s evenness index for each chain type, which reflects the evenness of the clone distributions within each sample (**Figure 2C**). This evenness index is calculated by dividing Shannon entropy by the maximum possible Shannon entropy index, essentially normalizing the Shannon entropy index by the number of unique clones in each sample. GBM, PCPG, and KICH have the highest evenness in all three chains while SKCM and TGCT have the lowest evenness compared to the other tumor types.

Next, we compared evenness between primary tumors and adjacent non-tumor samples across the Ig chains (**Figure 2D**). We found that evenness was significantly higher in adjacent non-tumor samples compared to tumors in 7/14 (BRCA, COAD, HNSC, KIRC, LIHC, LUAD, LUSC) tumor types for IGH, 5/14 (BRCA, HNSC, KIRC, LUAD, LUSC) for IGK, and 4/14 (BRCA, HNSC, KIRC, LUAD) for IGL. While evenness was consistently higher in adjacent non-tumor samples for IGH, we observed higher evenness in tumor samples compared to non-tumors in IGK and IGL for COAD.

### 3.3 Network Analysis Reveals Differences in Clonal Expansion and Diversification Across Tumor Types and Between Tumor and Adjacent Non-Tumor Samples

**Figure 3:**
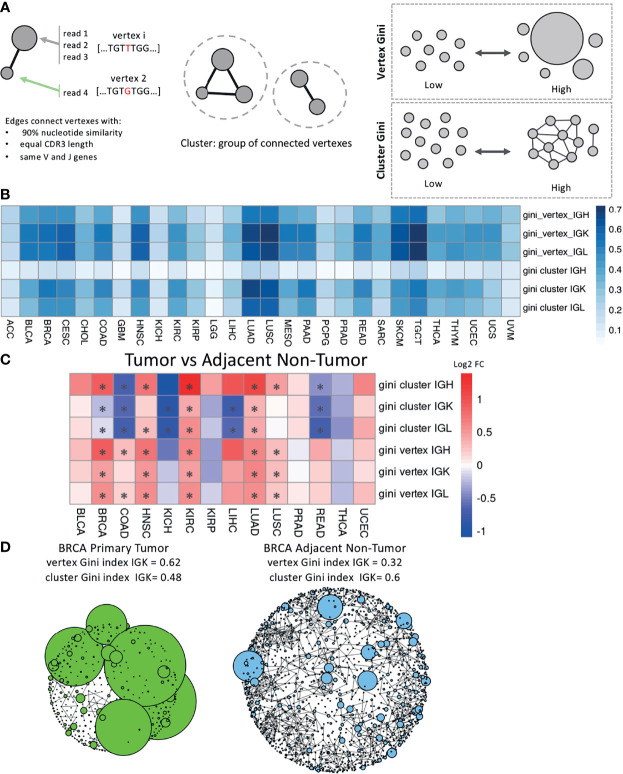
Network analysis across tumor types and between tumor and adjacent non-tumor samples. **(A)** Schematic describing how the networks were generated for each sample. The vertex Gini index and the cluster Gini index were used to quantify clonal expansion and clonal diversification. **(B)** A heatmap showing the mean vertex Gini index and cluster Gini index across tumor types in each chain. **(C)** A heatmap showing the log2 fold ratio between the mean vertex/cluster Gini index in tumor samples and the mean vertex/cluster Gini index in the adjacent non-tumor samples. Red indicates a higher mean value in tumors and blue indicates a higher mean value in adjacent non-tumor samples. Significance was computing using the Wilcoxon rank-sum test and the asterisks indicate an FDR < 0.05. **(D)** Network plots for a BRCA primary tumor sample on the left and a BRCA adjacent non-tumor sample on the right. Vertexes depict unique BCR sequences and sizes indicate the number of reads. Edges are drawn between vertexes that have the same V and J genes, the same CDR3 length, and at least 90% sequence similarity.

We generated networks for each sample using a previously published method (21, 22, 26) (**Figure 3A**). Each vertex in the network is a unique Ig sequence and the size of the vertex corresponds to the number of reads with that sequence. Edges connect vertexes that have the same V and J genes, the same CDR3 length, and at least 90% sequence similarity, and clusters are groups of connected vertexes. Clonal expansion of unique Ig sequences can be measured by calculating the Gini index of the vertex sizes. A high vertex Gini index indicates clonal expansion of unique Ig sequence(s) and a low Gini index indicates a more even distribution of vertex sizes. Clonal diversification can be measured by calculating the Gini index of the cluster sizes, which are the number of vertexes in each cluster. This measurement reflects the amount of diversification of B cell clones from SHM. A high cluster Gini index indicates a sample with unequal cluster sizes, which suggests that some clones are highly diversified, and a low cluster Gini index indicates a sample with even sized clusters.

We calculated the vertex and cluster Gini indexes on each sample’s network across the tumor types (**Figure 3B**). LUAD, LUSC, TGCT, and SKCM had the highest mean vertex Gini indexes across the Ig chains, indicating higher levels of clonal expansion in these tumor types. LUAD, LUSC, and TGCT also had the highest mean cluster Gini indexes across the Ig chains, suggesting high levels of clonal diversification in these tumor types. Interestingly, the cluster Gini indexes are lower in IGH compared to IGK and IGL across the tumor types, suggesting that IGH has lower clonal diversification. We also observed that ACC, GBM, LGG, and KICH have consistently lower Gini vertex and cluster indexes and little difference between the IGH, IGK and IGL chains, suggesting that the B cell repertoire may not be particularly active in these tumor types.

We then compared the vertex and cluster Gini indexes between tumor samples and adjacent non-tumor samples for the tumor types with at least 10 adjacent non-tumor samples (**Figure 3C**). The vertex Gini index was significantly higher in tumor samples in 6/14 tumor types for IGH, 5/14 for IGK, and 5/14 for IGL, and none of the tumor types had significantly higher vertex Gini indexes in the adjacent non-tumor samples compared to the tumor samples. This suggests that the tumor samples generally have higher clonal expansion compared to the adjacent non-tumor samples. Similarly, the cluster Gini indexes was higher in tumor samples compared to adjacent non-tumor samples in 5/14 tumor types for IGH. However, adjacent non-tumor samples had higher cluster Gini indexes in 5/14 tumor types for IGK and 5/14 tumor types for IGL. This suggests that there is a trend towards higher clonal diversification in tumor samples for IGH and a trend towards higher clonal diversification in adjacent non-tumor samples for IGK and IGL.

We generated plots for each sample and for each chain to visualize the networks. Example plots for BRCA tumor sample and an BRCA adjacent non-tumor sample are shown in **Figure 3D** with data for the IGK chain. The BRCA tumor sample has a higher vertex Gini index and more clonal expansion of individual reads, which can be seen in the large vertexes in the plot. The BRCA adjacent non-tumor sample has a higher cluster Gini index, which can be seen in the increased connectivity of some of the clusters in the plot.

Since the Gini indexes may be affected by differences in sequencing depth, we carried out a downsampling analysis similar to previous studies to confirm that our network analysis results are not driven by sequencing depth variability (21, 27, 28). We downsampled to 500 IGH, IGK, and IGL reads respectively and recalculated the vertex and cluster Gini indexes for each chain. Samples were removed if they did not have at least 500 reads in each chain, which removed a significant number of samples with low infiltration (**Supplementary Figure 3A**). The original analysis and the downsampled analysis were highly correlated for both vertex (*ρ* = 0.72-0.75) and cluster (*ρ* = 0.56-0.81) Gini indexes (**Supplementary Figure 3B**). The downsampled analysis comparing tumor and adjacent non-tumor samples also held the same general trends as the original analysis (**Supplementary Figure 3C**).

### 3.4 Case Study: Diversity and Network Analysis Across BRCA Subtypes

While our previous analysis made comparisons between tumor types, cancer is an incredibly heterogenous disease and each tumor type can often be divided into subtypes with different molecular characteristics and prognosis. We were interested in investigating the differences between tumor subtypes and we performed subtype-specific analysis for the following 19 tumor types with subtype information curated by TCGAbiolinks (20): ACC (29), BLCA (30), BRCA (31), COAD (32), GBM (33), HNSC (34), KICH (35), KIRC (36), KIRP (37), LGG (33), LIHC (38), LUAD (39), LUSC (40), PAAD (41), PCPG (42), PRAD (43), SKCM (44), THCA (45), UCEC (46). While 15/19 tumor types had significant differences between their subtypes (**Supplementary Figure 4**), we present the results for BRCA in the main text.

**Figure 4:**
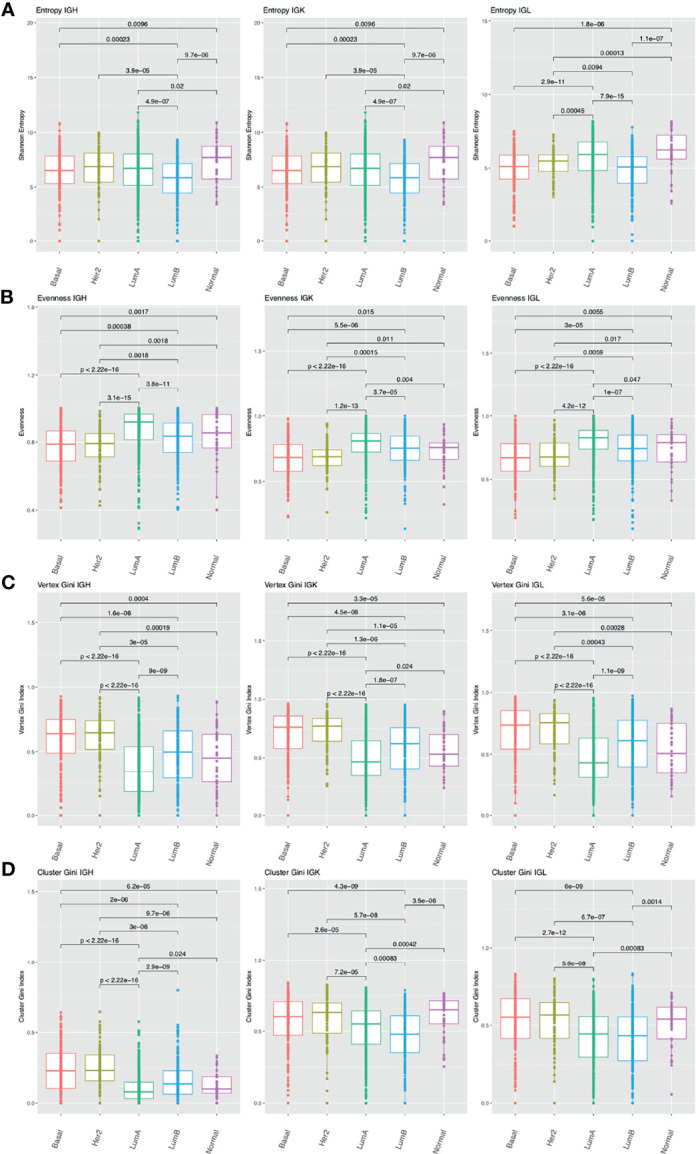
Differences in B cell repertoire features across breast cancer subtypes. **(A)** Boxplots depict the Shannon entropy index in each BRCA subtype for IGH, IGK, and IGL. Brackets indicate significant comparisons using the Wilcoxon rank-sum test and p-values are placed above each bracket. **(B)** Boxplots depict Pielou’s evenness index in each BRCA subtype. **(C)** Boxplots depict the vertex Gini index in each BRCA subtype. **(D)** Boxplots depict the cluster Gini index in each BRCA subtype.

Previous studies have shown that breast cancer can be divided into subtypes with different treatment responses and outcomes based (47, 48). These subtypes include: luminal A, luminal B, HER2-enriched, basal, and normal-like. Luminal A and normal-like breast cancers tend to have higher entropy compared to the other subtypes across the chain types (**Figure 4A**). The basal and HER2-enriched subtypes have lower evenness compared to the luminal A, luminal B, and normal-like subtypes across the chain types (**Figure 4B**). In the network analysis, the basal and the HER2-enriched subtypes have higher vertex Gini indexes across the chain types, indicating higher clonal expansion in these subtypes (**Figure 4C**). The basal and HER2-enriched subtypes also have higher cluster Gini indexes compared to the luminal subtypes across the chain types, indicating that there may be higher clonal diversification in these subtypes (**Figure 4D**). This analysis revealed differences in diversity, evenness, and network features across the BRCA subtypes.

### 3.5 B Cell Receptor Repertoire Features Associated With Host and Clinical Features and Mutation Load

We were interested in investigating associations between the B cell repertoire features and mutation load, which was defined as the number of non-silent mutations per megabase, as well as other host and clinical features available in TCGA (7, 49).

**Figure 5:**
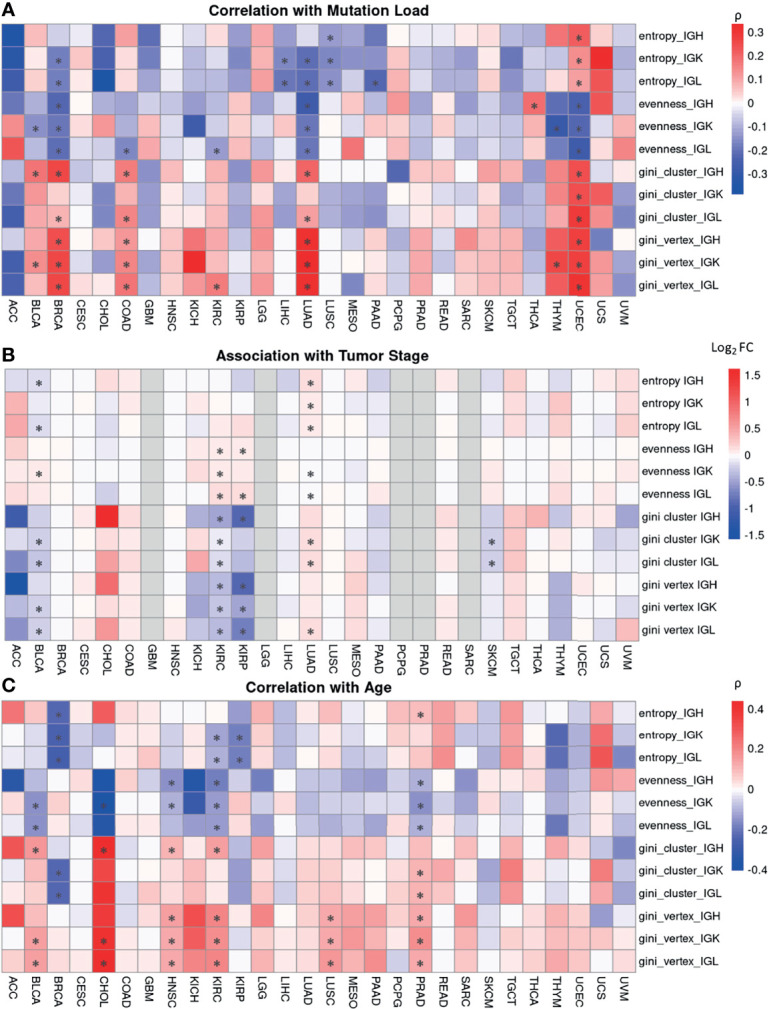
Associations between B cell repertoire features and tumor and clinical characteristics. **(A)** A heatmap depicting Spearman’s correlation coefficient for mutation load, which is the number of non-silent mutations per megabase, and each B cell repertoire feature. Significant correlations (FDR < 0.05) are marked by an asterisk. **(B)** A heatmap showing the log2 fold ratio between the mean of the stage I-II tumors and the mean of the stage III-IV tumors. Significance was computing using the Wilcoxon rank-sum test and the asterisks indicate an FDR < 0.05. **(C)** A heatmap depicting Spearman’s correlation coefficient between patient’s age at diagnosis and each B cell repertoire feature. Significant correlations (FDR < 0.05) are marked by an asterisk.

First, we correlated the B cell repertoire features with mutation load and found that mutation load was not significantly correlated with immune features in a majority of the tumor types analyzed. We also observed that the tumor types with significant correlations between their repertoire features and mutation load were not necessarily the tumor types with the highest mutational loads overall (**Supplementary Figure 5A**). However, in the tumor types with significant correlations, mutation load seems to be largely negatively correlated with entropy and evenness (**Figure 5A**). This suggests that having a more diverse, even B cell repertoire seems to be associated with tumors that have lower mutation load. The exceptions were UCEC, which had a positive correlation between mutation load and entropy, and THCA, which had a positive correlation between mutation load and evenness. Similarly, when comparing mutation load to the vertex and cluster Gini indexes, few tumor types had significant correlations. However, among the tumor types with significant correlations, mutation load was positively correlated with the vertex and cluster Gini indexes. This suggests that higher clonal expansion and higher clonal diversification is associated with higher mutation load, perhaps because tumors with higher mutation loads can generate more neoantigens which can drive a better immune response. Indeed, previous studies have shown that a higher non-synonymous mutation burden in tumors was associated with improved response to immunotherapies (50, 51).

We were also interested in investigating associations between the B cell repertoire features and tumor stage. We compared the lower stage tumors (Stage I-II) to higher stage tumors (III-IV) and found that there was no significant difference in a majority of the tumor types (**Figure 5B**). In the 5 tumor types with significant associations, having a higher tumor stage was associated with higher vertex and cluster Gini indexes in 4/5 tumor types, suggesting that there may be slightly increased clonal expansion and clonal diversification in tumors with higher stages.

Next, we correlated age at diagnosis with the B cell repertoire features and found significant associations in 8 tumor types (**Figure 5C**). Age at diagnosis was negatively correlated with Shannon entropy in BRCA, KIRC, and KIRP, similar to a previous study (52). We also found a negative correlation between age and evenness and a positive correlation between age and the vertex Gini indexes in BLCA, HNSC, KIRC, and PRAD. The overall correlation strengths were relatively low, suggesting a possible slight increase of clonal expansion in older patients.

Lastly, we compared the B cell repertoire features between sexes (**Supplementary Figure 5B**) and found very few significant associations across tumor types and repertoire features. Females have significantly higher entropy than males in BLCA but the log fold difference between the mean female entropy value and the mean male entropy value was relatively small.

Overall, the B cell repertoire features were not significantly associated with mutation load or clinical features in a majority of the tumor types. However, there did seem to be some consistent trends among the tumor types with significant associations, suggesting that there may be a subtle signal in these tumor types.

### 3.6 B Cell Repertoire Features Are Prognostic in Select Tumor Types

**Figure 6:**
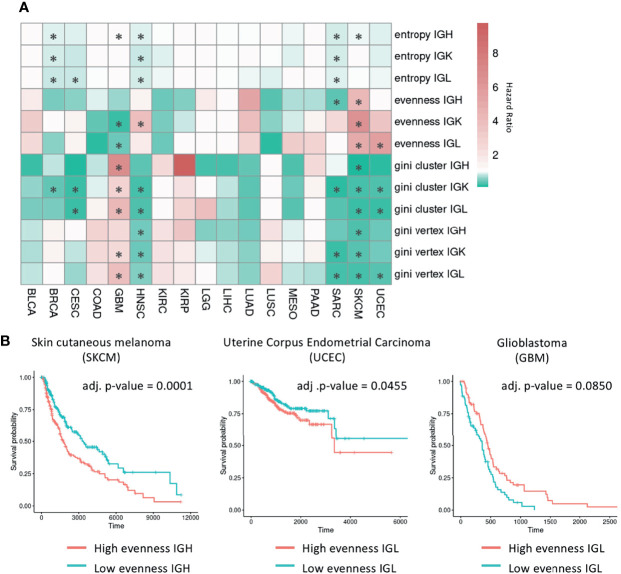
Survival analysis using B cell repertoire features. **(A)** Heatmap showing the hazard ratio from a Cox proportional hazards model for each B cell repertoire feature adjusted for age, gender, and tumor stage. Red indicates a hazard ratio greater than 1 and green indicates a hazard ratio less than 1. Significant associations (FDR < 0.1) are marked by an asterisk. **(B)** Kaplan-Meier curves for samples with high and low evenness with the median evenness value used as the cutoff. The adjusted p-values are from the multivariate Cox regression models adjusted for age, gender, and tumor stage with the Benjamini-Hochberg procedure used to control for multiple comparisons.

We built Cox proportional hazard models for each B cell repertoire feature to investigate associations with survival while adjusting for age, gender, and tumor stage (**Figure 6A** and **Supplementary Table 2**). We selected tumor types with at least 40 events to have at least 10 events per predictor variable (53). After FDR correction, we found significant associations (FDR < 0.1) in 7/17 of the tumor types analyzed.

In the six tumor types that have significant associations with Shannon entropy (BRCA, CESC, GBM, HNSC, SARC, SKCM), having a higher entropy value was associated with improved survival in all tumor types except GBM. In the five tumor types that have significant associations with evenness, having a higher evenness was associated with improved survival in SARC and GBM for a subset of chains. However, a higher evenness was associated with decreased survival in the IGK chain for HNSC, in the IGL chain for UCEC, and across all chains in SKCM as seen in a previous study (54) (**Figure 6B**). This suggests that B cells may be playing different roles in these tumor types. Vertex and cluster Gini indexes were significantly associated with survival in at least one chain type in BRCA, CESC, GBM, HNSC, SARC, SKCM, and UCEC. In BRCA, CESC, HNSC, SARC, SKCM, and UCEC, having a higher vertex and cluster Gini indexes was associated with improved survival, suggesting clonal expansion may be beneficial in these tumor types. However, having a higher vertex Gini index was associated with worse survival in GBM, suggesting that clonal expansion may be detrimental in this tumor type.

We also stratified the tumors by subtype and repeated the analysis to see if specific subtypes reveal different behaviors (**Supplementary Figure 6**). While five tumor types (HNSC, KIRC, LGG, LUSC, SKCM) had at least one subtype with significant associations with survival, we did not observe differences between subtypes from the same tumor type.

### 3.7 V-Gene Usage Reveals Similarities in COAD, READ, and UCEC Repertoires

**Figure 7:**
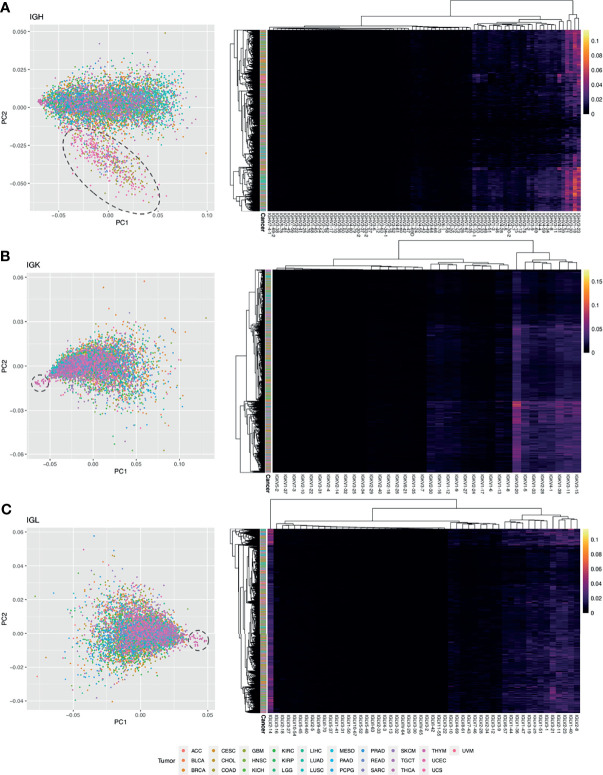
Analysis of V gene usage. **(A)** PCA plot using IGH V gene usage data. Each point is a sample and the color of the point corresponds to a tumor type. A dashed circle is drawn around a cluster of COAD, READ, and UCEC samples. On the right is a heatmap with hierarchical clustering performed on the samples and the IGH V gene usage data. The intensity of the heatmap corresponds to the percent of clones using each V gene. **(B)** PCA plot using IGK V gene usage data. A dashed circle is drawn around a cluster of THYM samples. On the right is a heatmap with hierarchical clustering performed on the samples and the IGK V gene usage data. **(C)** PCA plot using IGL V gene usage data. A dashed circle is drawn around a cluster of THYM samples. On the right is a heatmap with hierarchical clustering performed on the samples and the IGL V gene usage data.

Previous studies have shown that V-gene usage may differ in tumor tissues (55). We wanted to investigate differences in V-gene usage across the tumor types analyzed in this study. We defined V-gene usage here as the percent of clones in each sample that use a particular V-gene. We then used principal component analysis (PCA) to reduce the dimensionality of the V-gene usage data and plotted the first two principal components to visualize the data for each Ig chain (**Figures 7A–C**).

For the IGH analysis, we used k-means clustering to identify two clusters in the data after dimensionality reduction with PCA (**Supplementary Figure 7A**). One cluster was primarily comprised of COAD, READ, and UCEC samples (**Supplementary Figure 7B**), suggesting that these samples have a similar V gene usage pattern. We used an elastic net model to identify the V genes that are associated with this cluster and identified 45 V-genes (**Supplementary Figure 7C**). We also performed hierarchical clustering on the IGHV gene usage data (**Figure 7A**) and found a cluster of four V genes that have relatively high usage compared to the others (IGHV3-21, IGHV3-23, IGH30-30, IGHV1-18), which is consistent with previous studies (56, 57).

Next, we performed a similar analysis for the IGK and IGL chains. We identified a group of 19 THYM samples that separated out from the other tumor samples in PC1 for both IGK and IGL (**Figures 7B, C**). These samples had significantly lower V-gene usage (Wilcoxon rank-sum test IGK p-value = 2.8e-06, IGL p-value = 3.9e-05) compared to the other samples, although we could not find significant associations with clinical features such as tumor site or having a history of myasthenia gravis, which is an autoimmune neuromuscular disease found in 50% of cortical thymoma patients (58). We used an elastic net model to identify the V-genes that are associated with these THYM samples and we identified 24 V IGK V-genes (**Supplementary Figure 7D**) and 44 IGL V-genes (**Supplementary Figure 7E**). After performing hierarchical clustering on the IGKV gene usage data, we identified a cluster of eight IGKV genes (IGKV3-20, IGKV1-5, IGKV1-33, IGKV2-28, IGKV4-1, IGKV1-39, IGKV3-11, IGKV3-15) with relatively high usage compared to the other V-genes. Similarly, the hierarchical clustering results for IGL identified a cluster of 13 IGL V-genes with relatively high usage (IGLV6-57, IGLV1-44, IGLV1-36, IGLV1-47, IGLV3-19, IGLV3-25, IGLV1-51, IGLV3-1, IGLV3-21, IGLV2-11, IGLV2-23, IGLV1-40, IGLV2-8). Interestingly, the IGLV2-14 V-gene formed its own cluster separate from every other IGL V-gene and seems to have relatively high usage across many tumor samples (**Figure 7C**).

## Discussion

Many studies have established the importance of T cells in immunosurveillance and immunotherapy response in cancer. However, the role of B cells has not been as well studied and tumor-infiltrating B cells have been shown to have both protumor and antitumor effects (1). Current bioinformatic tools allowed us to interrogate the composition of B cell repertoires from RNA-seq data through a pan-cancer approach, offering more detailed insights into the B cell response to tumors. For example, Thorsson et al. extracted and analyzed the TCR and BCR repertoires from the TCGA RNA-seq dataset, but their analysis did not include tumor subtype stratification, comparisons between the BCR repertoires in tumor versus adjacent non-tumor tissue, or associations between BCR repertoire features and clinical features. In this study, we analyzed the B cell repertoires of 9,522 tumor and adjacent non-tumor samples across 28 tumor types and their subtypes using TCGA RNA-seq and clinical data.

All tumor samples were assessed for immune repertoire features, including Ig expression, Shannon entropy, clonal expansion and clonal diversification, which revealed large differences among the different tumor types. We found the highest expression of IGH, IGK, and IGL chains in LUSC and LUAD, which is similar to previous studies which found an abundant and diverse B cell population in non-small cell lung cancers (59). Many of the tumor types with the highest Ig chain expression also have the highest overall leukocyte fraction and are most responsive to checkpoint inhibitors (7), suggesting that B cells may help promote response to immunotherapies. Indeed, several studies have shown that an enrichment of B cells in tertiary lymphoid structures was predictive of response to immune checkpoint inhibitors in melanoma, soft-tissue sarcoma, and renal cell carcinoma (60, 61). Moreover, we also found that the tumor types with low IGH, IGK and IGL expression, such as UVM and ACC, also have low overall leukocyte fraction and poor responses to immunotherapies (23, 24). These patterns were also observed in the diversity analysis using Shannon entropy, which was unsurprising given the overall Ig expression in these tumors, and the vertex and cluster Gini index indicating a clear clonal expansion and diversification in the tumors with high overall Ig expression and entropy.

We also found a significant positive correlation between expression and Shannon entropy in all the tumor types analyzed, similar to previous studies (54). Interestingly, we found that the Shannon entropy indexes in IGK and IGL had lower correlations with expression compared to IGH and that the cluster Gini indexes for IGH were lower than IGK and IGL. This suggests that there may be more uneven distribution of clones in IGK and IGL compared to IGH, which is reflected in IGK and IGL having lower Shannon entropy despite having higher expression than IGH. Indeed, IGK and IGL are less diverse since they are produced by recombination of only the V and J genes and they likely need to go through clonal expansion and somatic hypermutation after exposure to an antigen.

Differences in B cell repertoire between tumor and adjacent non-tumor samples may be due to response to tumor specific antigens. The tumor samples tend to have lower Shannon entropy and vertex Gini indexes compared to primary tumor samples, suggesting that the increased clonal expansion in the tumor samples may be the result of B cells in the tumor microenvironment reacting to tumor neoantigens.

In our tumor subtype analysis, we analyzed 19 tumor types and found significant differences between subtypes in 15 tumor types. We decided to focus on BRCA as a case study in the main text. The basal and HER2-enriched subtypes have lower evenness and higher vertex Gini indexes and cluster Gini indexes, suggesting more clonal expansion in these subtypes. Interestingly, previous studies have shown that the basal and HER2-enriched subtypes tend to have high immune infiltration (62, 63) and were the only subtypes where increased expression of B cell signatures was associated with metastasis-free survival (64).

Although we found large differences among the tumor types with their B cell repertoire, we found that few tumor types had significant associations between their B cell repertoire features and mutation load, tumor stage, and age. Among the tumor types with significant associations, we found that the repertoire features associated with higher clonal expansion and clonal diversification were positively correlated with mutation load. This is in line with previous studies which have shown that a higher mutation burden is associated with improved immunotherapy responses (50, 51). We also found that age tends to be negatively correlated with evenness and positively correlated with vertex and cluster Gini indexes, suggesting that older patients have greater clonal expansion than younger patients. This reflects previous observations of decreased B cell diversity and increased clonal expansion in normal aging (65). Overall, the B cell repertoire features were not significantly associated with mutation load or clinical features in a majority of the tumor types. However, there did seem to be some consistent trends among the tumor types with significant associations, suggesting that there may be a subtle signal in these tumor types.

Next, we built Cox proportional hazard models for each B cell repertoire feature to investigate the prognostic value of each feature. After adjusting for age, gender, and tumor stage, we were unable to find significant associations for a majority of the tumor types analyzed. However, in the tumor types with significant associations, we found some opposing trends such as evenness being associated with decreased survival in SKCM and UCEC but increased survival in GBM and SARC. Previous studies have shown that B cells can differentiate into plasmablast-like cells in SKCM (66) while they may act primarily as antigen-presenting cells in GBM (67), supporting the idea that B cells play different roles in these tumor types.

Finally, our V-gene usage analysis revealed that a subset of COAD, READ, and UCEC samples have similar IGH V gene usage patterns. Interestingly, a recent study that predicted tumor type from BCR sequences found that COAD samples were likely to be predicted as UCEC in their model, supporting the idea that these tumor types may have similar B cell repertoires (68). We also identified a subset of THYM patients with overall low V-gene usage, although we could not find significant associations between this subset of patients and any clinical variables. In our hierarchical clustering analysis, we identified clusters of V-genes in each chain that had higher overall usage across the tumor types which were largely consistent with previous studies (56, 57). For the IGL V-gene analysis, we found that the IGLV2-14 V-gene formed its own cluster and seems to have relatively high usage across many tumor samples. Interestingly, IGLV2-14 has been previously associated with chronic lymphocytic leukemia (69), multiple melanoma (70), and it is the most common IGLV gene in human cord blood (71).

There are several limitations that should be noted. One limitation of our study is the use of RNA-seq data rather than targeted sequencing data (e.g. BCR-seq). While RNA-seq data has lower sequencing depth compared to targeted sequencing data, we chose to use RNA-seq data because we wanted to leverage the large number of tumor samples in the TCGA dataset. Additionally, it is not possible to perform paired heavy and light chain analysis using short-read bulk RNA-seq data. Therefore, results from this study warrants validation with targeted sequencing data. Another limitation of this study is the limited number of adjacent non-tumor samples and the lack of true healthy samples, as previous studies have shown that adjacent non-tumor samples tend to have more inflammatory-associated cell types compared to healthy samples (72). Additional analysis using datasets with both tumor and healthy samples would be informative for validating the results of this study. Moreover, the TCGA dataset, like many adaptive immune receptor repertoire sequencing (AIRR-seq) datasets (73), is primarily comprised of individuals with European ancestry (74). Including more individuals with non-European ancestry in immunogenomic studies is critical to understanding population differences in the adaptive immune system and improving precision immunodiagnostics and therapeutics. Lastly, our study is unable to perform analysis within individual types of B cells, which single-cell sequencing would allow, or to analyze the localization of B cell populations within the tumor, which new technologies such as spatial transcriptomics would allow. However, the amount of data generated using these newer technologies is limited compared to the amount of publicly available RNA-seq data currently available, making it more feasible for future studies.

In summary, our study characterizes the B cell repertoire of 28 tumor types and reveals differences across tumors and tumor subtypes, as well as between adjacent non-tumor and tumor samples. These results help further our understanding of the role of B cells in the tumor microenvironment with implications for the development of novel B cell immunotherapies, therapeutic strategies, and patient stratification.

## Data Availability Statement

Publicly available datasets were analyzed in this study. This data can be found here: https://portal.gdc.cancer.gov/legacy-archive/search/f.

## Author Contributions

KY and SP performed the data analysis. KY and AR extracted the data. KY, SP, and MS wrote the paper. KY, SP, MS, and NM reviewed and edited the paper. SP, MS, and NM supervised the work. All authors contributed to the article and approved the submitted version.

## Funding

This work was supported by the 2019 AACR-AstraZeneca Immuno-oncology Research Fellowship (19-40-12-PINE). The funder was not involved in the study design, collection, analysis, interpretation of data, the writing of this article or the decision to submit it for publication.

## Conflict of Interest

The authors declare that the research was conducted in the absence of any commercial or financial relationships that could be construed as a potential conflict of interest.

## Publisher’s Note

All claims expressed in this article are solely those of the authors and do not necessarily represent those of their affiliated organizations, or those of the publisher, the editors and the reviewers. Any product that may be evaluated in this article, or claim that may be made by its manufacturer, is not guaranteed or endorsed by the publisher.
