# Supplementary material for: A Pan-Cancer Analysis of Tumor-Infiltrating B Cell Repertoires
Source: Front Immunol. 2022 Jan 5;12:790119. doi: 10.3389/fimmu.2021.790119 (PMC8767103; doi:10.3389/fimmu.2021.790119)
Supplement: Supplementary file 1 [file DataSheet_1.pdf]

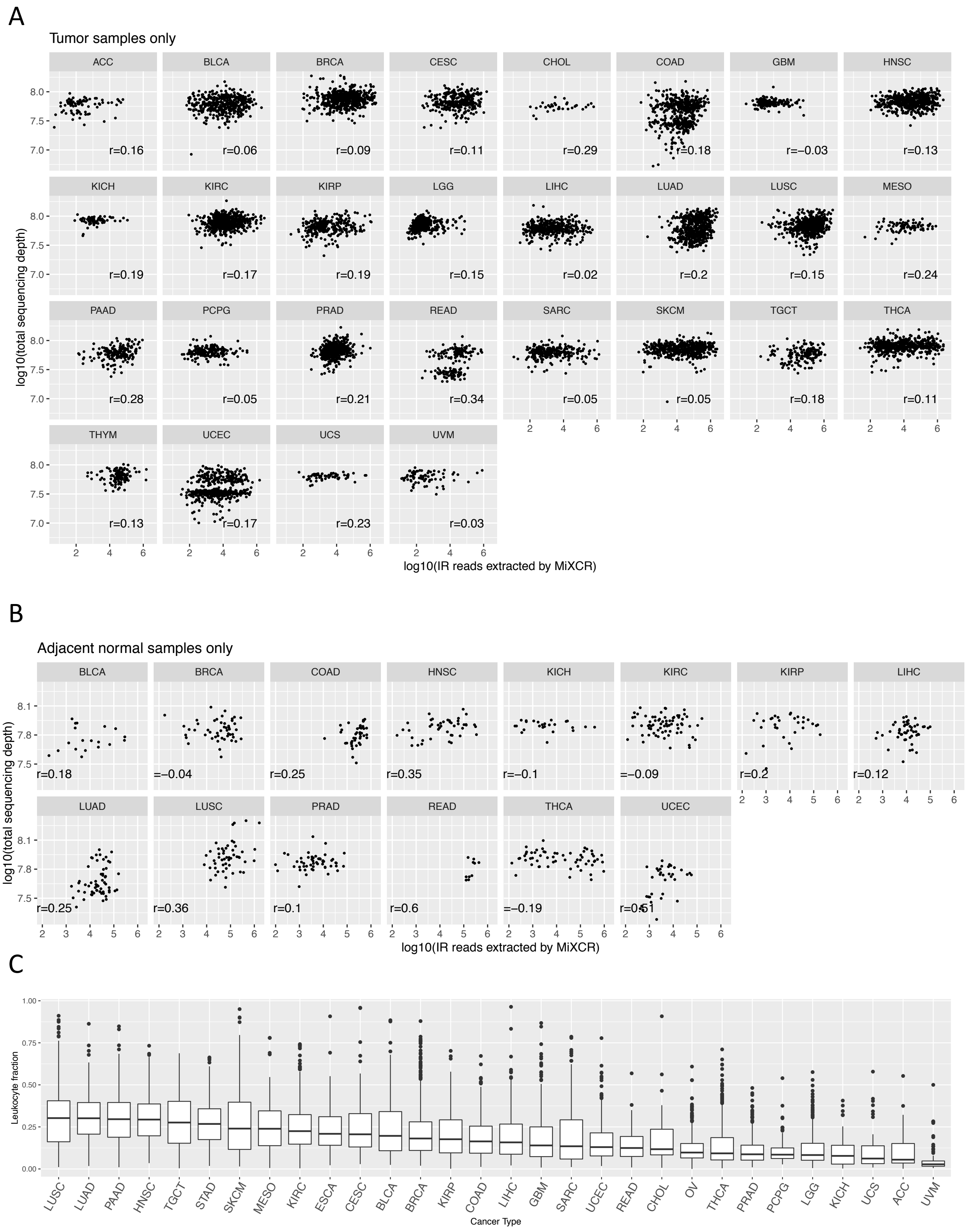

Supplementary Figure 1. Sample QC. (a) Plot showing the number of BCR reads extracted by MiXCR (x-axis) and the total number of reads in each sample (y-axis) for the tumor samples in each tumor type. (b) Plot showing the number of BCR reads extracted by MiXCR (x-axis) and the total number of reads in each sample (y-axis) for the adjacent normal samples in each tumor type (only tumor types with at least 10 adjacent normal samples are shown). (c) Boxplots showing the leukocyte fraction of each sample across tumor types. Tumor types are ordered by median leukocyte fraction from high to low.

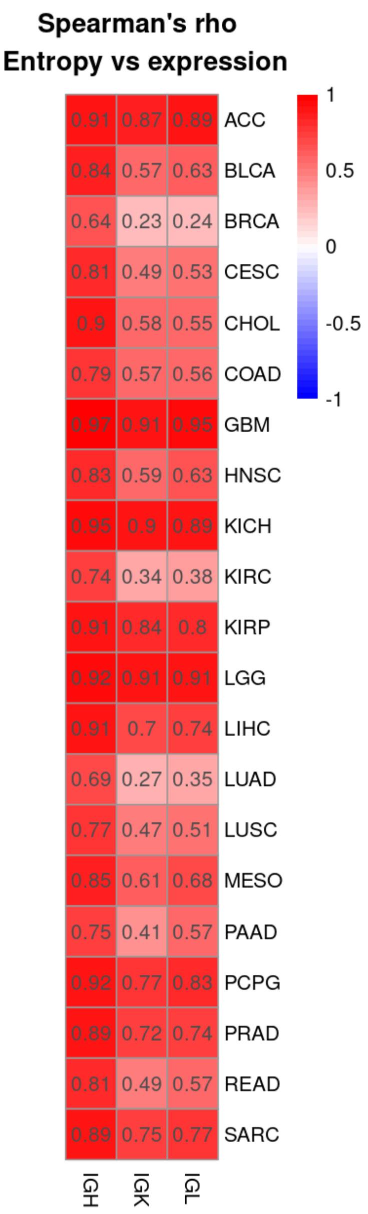

Supplementary Figure 2. Heatmap showing Spearman's correlation coefficient for Shannon entropy and expression (e.g. the number of IGH/IGK/IGL reads divided by the total number of reads in the sample). The value in each cell is the correlation coefficient.

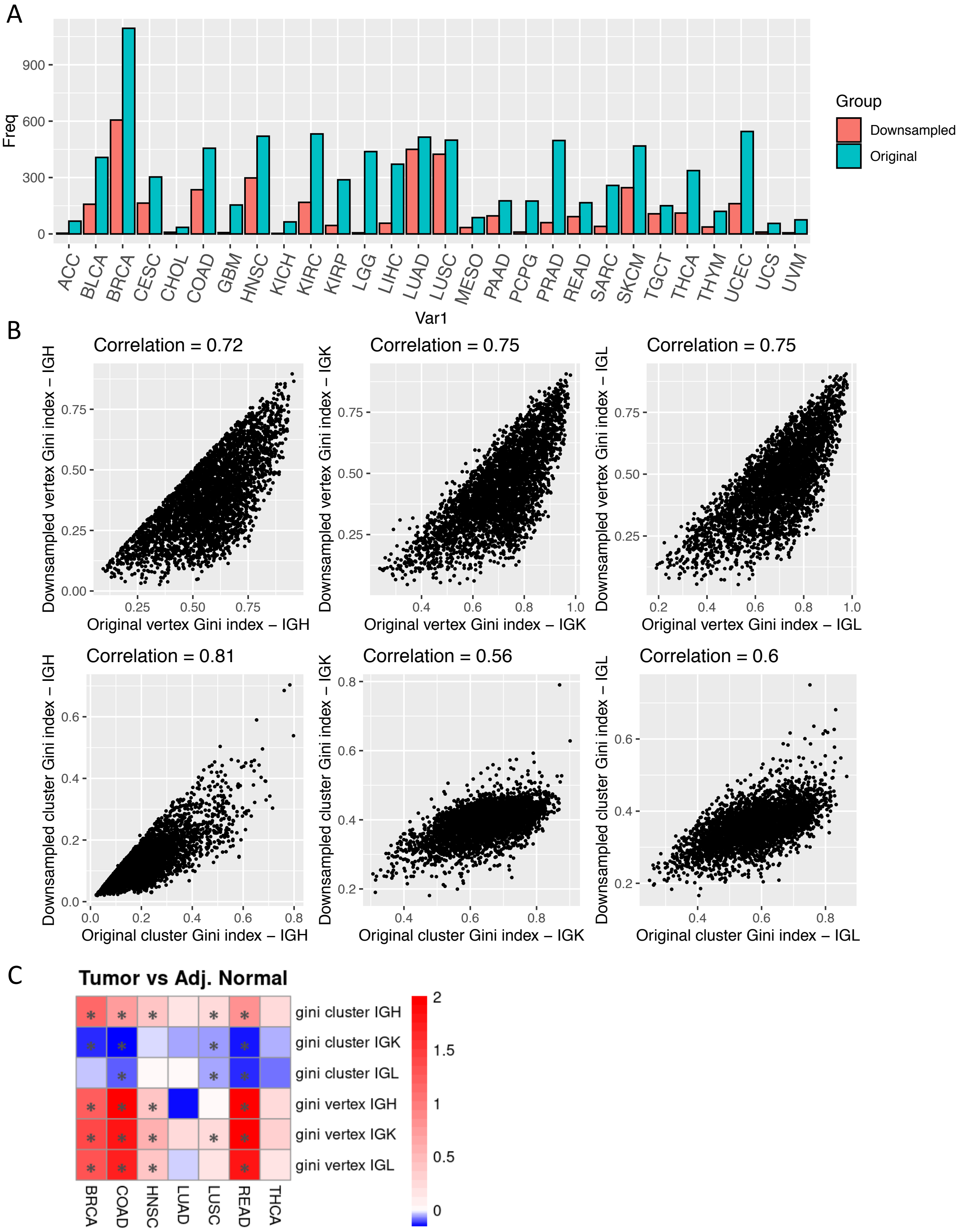

Supplementary Figure 3. Downsampling analysis for Gini vertex and Gini cluster measurements. (a) Barplot showing the number of samples used in the original analysis (turqoise) and in the downsampled analysis (red). The downsampled analysis removed samples with fewer than 500 IGH, IGK, and IGL reads. (b) Plots showing the original vertex or cluster Gini indexes (x-axis) versus the downsampled indexes (y-axis). (c). Heatmap showing the log2 fold ratio between the mean tumor value and the mean adjacent normal value. Statistical significance was calculated using the Wilcoxon rank-sum test and comparisons with FDR < 0.05 are marked by an asterisk.

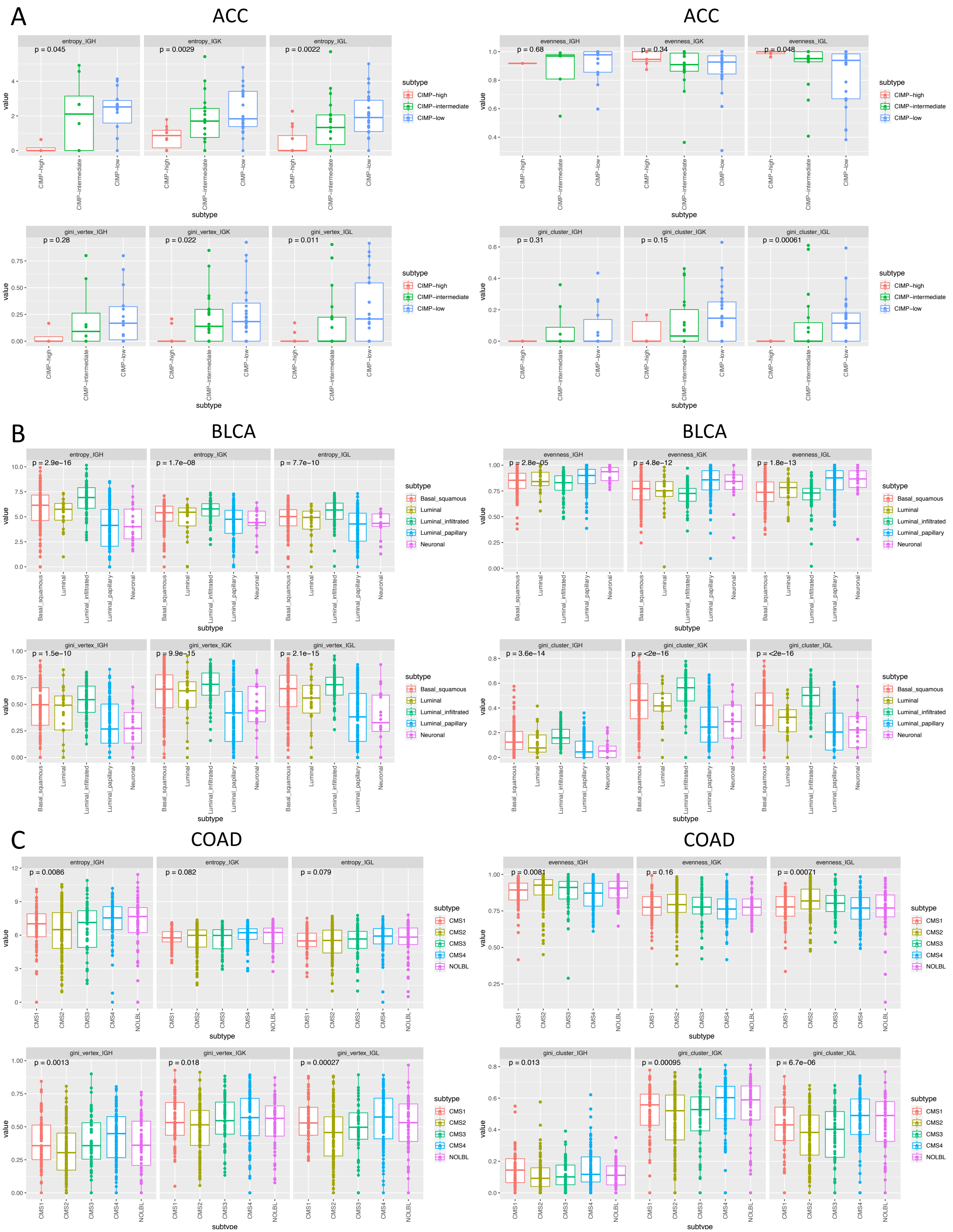

A
ACC
entropy_IGH
p = 0.045
entropy_IGK
p = 0.0029
entropy_IGL
p = 0.0022
evenness_IGH
p = 0.68
evenness_IGK
p = 0.34
evenness_IGL
p = 0.048
gini_vertex_IGH
p = 0.28
gini_vertex_IGK
p = 0.022
gini_vertex_IGL
p = 0.011
gini_cluster_IGH
p = 0.31
gini_cluster_IGK
p = 0.15
gini_cluster_IGL
p = 0.00061
value
subtype
CIMP−high
CIMP−intermediate
CIMP−low
B
BLCA
entropy_IGH
p = 2.9e−16
entropy_IGK
p = 1.7e−08
entropy_IGL
p = 7.7e−10
evenness_IGH
p = 2.8e−05
evenness_IGK
p = 4.8e−12
evenness_IGL
p = 1.8e−13
gini_vertex_IGH
p = 1.5e−10
gini_vertex_IGK
p = 9.9e−15
gini_vertex_IGL
p = 2.1e−15
gini_cluster_IGH
p = 3.6e−14
gini_cluster_IGK
p = <2e−16
gini_cluster_IGL
p = <2e−16
Basal_squamous
Luminal
Luminal_infiltrated
Luminal_papillary
Neuronal
C
COAD
entropy_IGH
p = 0.0086
entropy_IGK
p = 0.082
entropy_IGL
p = 0.079
evenness_IGH
p = 0.0081
evenness_IGK
p = 0.16
evenness_IGL
p = 0.00071
gini_vertex_IGH
p = 0.0013
gini_vertex_IGK
p = 0.018
gini_vertex_IGL
p = 0.00027
gini_cluster_IGH
p = 0.013
gini_cluster_IGK
p = 0.00095
gini_cluster_IGL
p = 6.7e−06
CMS1
CMS2
CMS3
CMS4
NOLBL

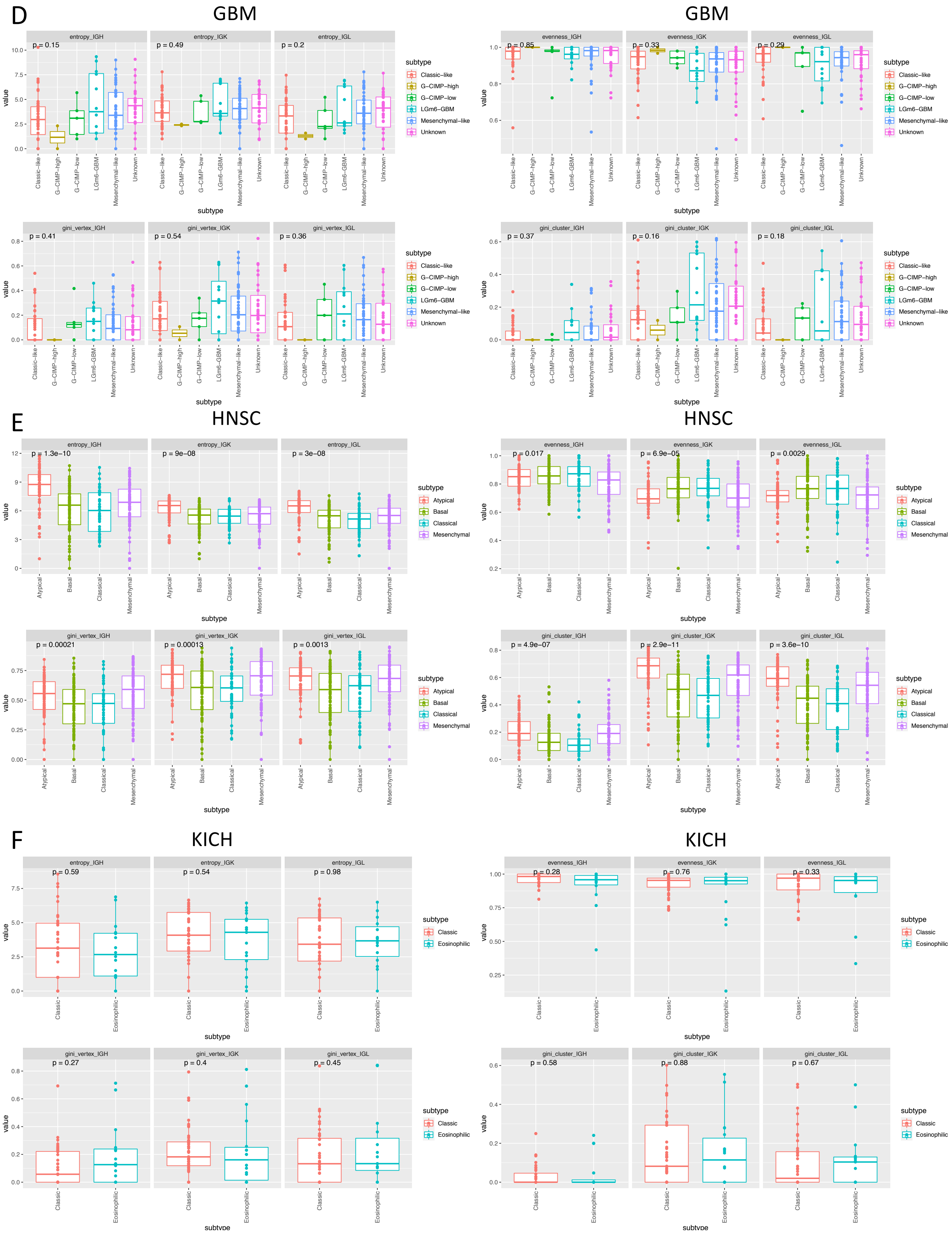
D
GBM
entropy_IGH
p = 0.15
entropy_IGK
p = 0.49
entropy_IGL
p = 0.2
evenness_IGH
p = 0.85
evenness_IGK
p = 0.33
evenness_IGL
p = 0.29
gini_vertex_IGH
p = 0.41
gini_vertex_IGK
p = 0.54
gini_vertex_IGL
p = 0.36
gini_cluster_IGH
p = 0.37
gini_cluster_IGK
p = 0.16
gini_cluster_IGL
p = 0.18
value
subtype
Classic−like
G−CIMP−high
G−CIMP−low
LGm6−GBM
Mesenchymal−like
Unknown
E
HNSC
entropy_IGH
p = 1.3e−10
entropy_IGK
p = 9e−08
entropy_IGL
p = 3e−08
evenness_IGH
p = 0.017
evenness_IGK
p = 6.9e−05
evenness_IGL
p = 0.0029
gini_vertex_IGH
p = 0.00021
gini_vertex_IGK
p = 0.00013
gini_vertex_IGL
p = 0.0013
gini_cluster_IGH
p = 4.9e−07
gini_cluster_IGK
p = 2.9e−11
gini_cluster_IGL
p = 3.6e−10
value
subtype
Atypical
Basal
Classical
Mesenchymal
F
KICH
entropy_IGH
p = 0.59
entropy_IGK
p = 0.54
entropy_IGL
p = 0.98
evenness_IGH
p = 0.28
evenness_IGK
p = 0.76
evenness_IGL
p = 0.33
gini_vertex_IGH
p = 0.27
gini_vertex_IGK
p = 0.4
gini_vertex_IGL
p = 0.45
gini_cluster_IGH
p = 0.58
gini_cluster_IGK
p = 0.88
gini_cluster_IGL
p = 0.67
value
subtype
Classic
Eosinophilic

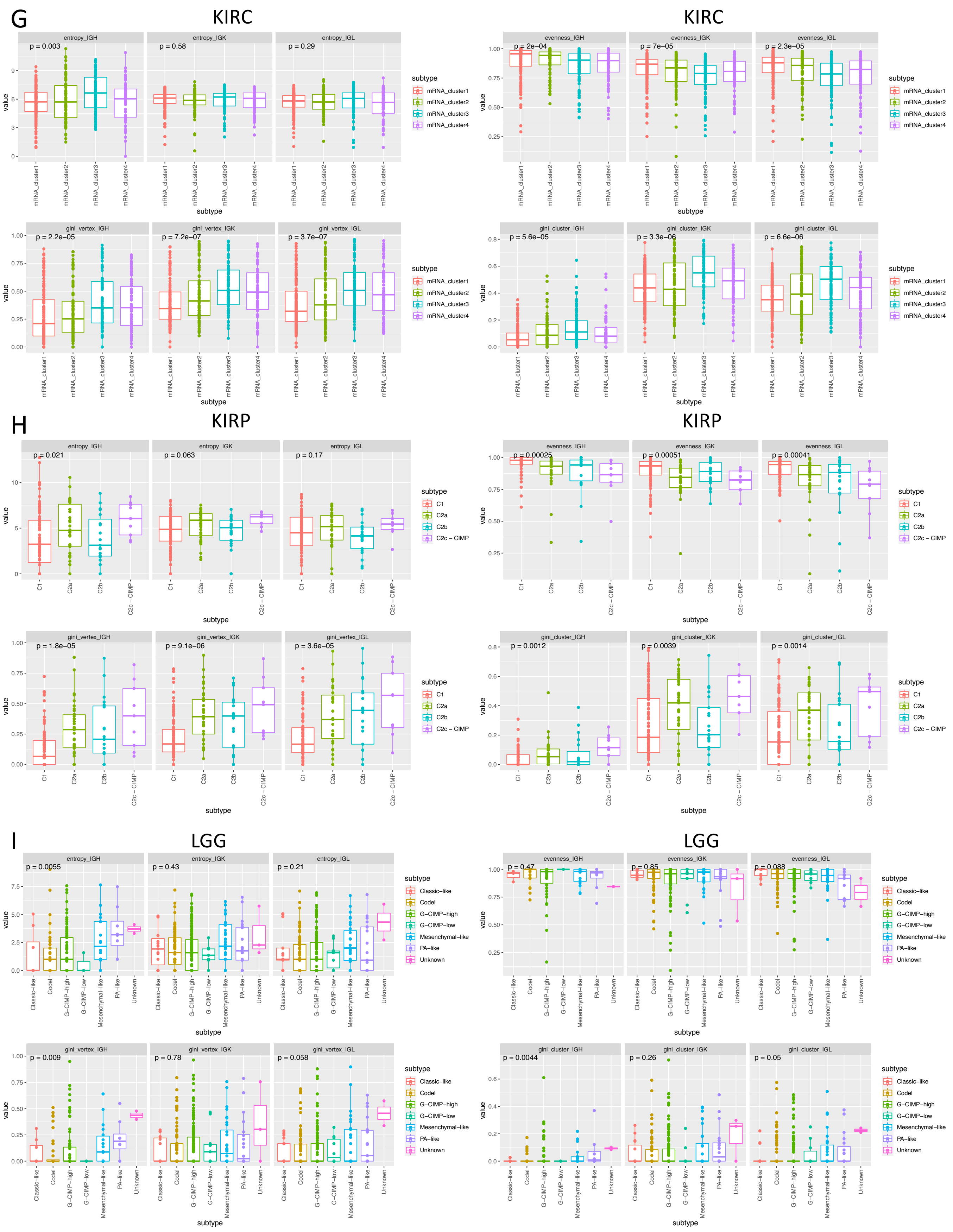

G
KIRC
entropy_IGH
p = 0.003
entropy_IGK
p = 0.58
entropy_IGL
p = 0.29
evenness_IGH
p = 2e−04
evenness_IGK
p = 7e−05
evenness_IGL
p = 2.3e−05
gini_vertex_IGH
p = 2.2e−05
gini_vertex_IGK
p = 7.2e−07
gini_vertex_IGL
p = 3.7e−07
gini_cluster_IGH
p = 5.6e−05
gini_cluster_IGK
p = 3.3e−06
gini_cluster_IGL
p = 6.6e−06
subtype
mRNA_cluster1
mRNA_cluster2
mRNA_cluster3
mRNA_cluster4
value
H
KIRP
entropy_IGH
p = 0.021
entropy_IGK
p = 0.063
entropy_IGL
p = 0.17
evenness_IGH
p = 0.00025
evenness_IGK
p = 0.00051
evenness_IGL
p = 0.00041
gini_vertex_IGH
p = 1.8e−05
gini_vertex_IGK
p = 9.1e−06
gini_vertex_IGL
p = 3.6e−05
gini_cluster_IGH
p = 0.0012
gini_cluster_IGK
p = 0.0039
gini_cluster_IGL
p = 0.0014
subtype
C1
C2a
C2b
C2c − CIMP
I
LGG
entropy_IGH
p = 0.0055
entropy_IGK
p = 0.43
entropy_IGL
p = 0.21
evenness_IGH
p = 0.47
evenness_IGK
p = 0.85
evenness_IGL
p = 0.088
gini_vertex_IGH
p = 0.009
gini_vertex_IGK
p = 0.78
gini_vertex_IGL
p = 0.058
gini_cluster_IGH
p = 0.0044
gini_cluster_IGK
p = 0.26
gini_cluster_IGL
p = 0.05
subtype
Classic−like
Codel
G−CIMP−high
G−CIMP−low
Mesenchymal−like
PA−like
Unknown

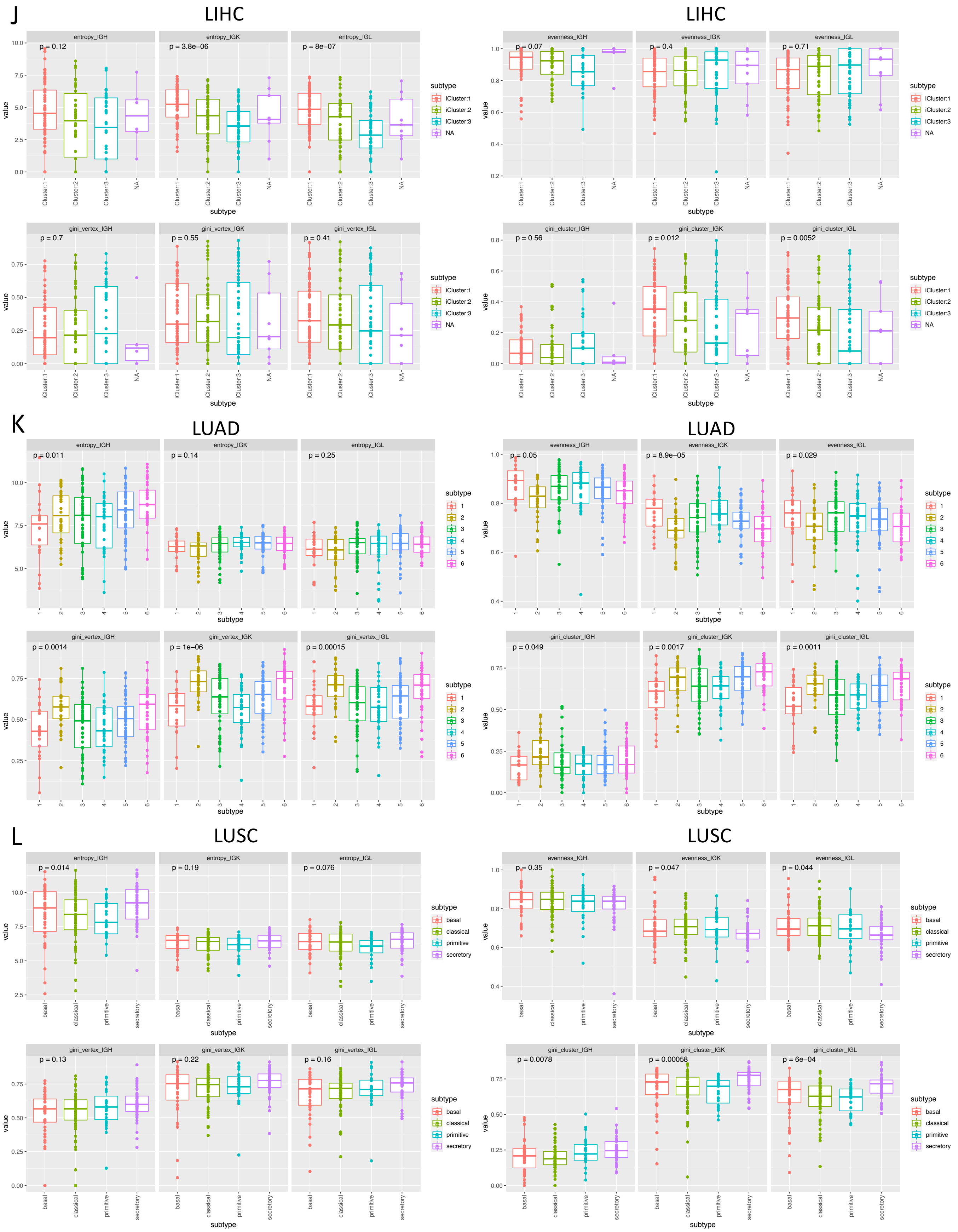

J
LIHC
entropy_IGH
p = 0.12
entropy_IGK
p = 3.8e−06
entropy_IGL
p = 8e−07
evenness_IGH
p = 0.07
evenness_IGK
p = 0.4
evenness_IGL
p = 0.71
gini_vertex_IGH
p = 0.7
gini_vertex_IGK
p = 0.55
gini_vertex_IGL
p = 0.41
gini_cluster_IGH
p = 0.56
gini_cluster_IGK
p = 0.012
gini_cluster_IGL
p = 0.0052
subtype
iCluster:1
iCluster:2
iCluster:3
NA
value
K
LUAD
entropy_IGH
p = 0.011
entropy_IGK
p = 0.14
entropy_IGL
p = 0.25
evenness_IGH
p = 0.05
evenness_IGK
p = 8.9e−05
evenness_IGL
p = 0.029
gini_vertex_IGH
p = 0.0014
gini_vertex_IGK
p = 1e−06
gini_vertex_IGL
p = 0.00015
gini_cluster_IGH
p = 0.049
gini_cluster_IGK
p = 0.0017
gini_cluster_IGL
p = 0.0011
subtype
1
2
3
4
5
6
L
LUSC
entropy_IGH
p = 0.014
entropy_IGK
p = 0.19
entropy_IGL
p = 0.076
evenness_IGH
p = 0.35
evenness_IGK
p = 0.047
evenness_IGL
p = 0.044
gini_vertex_IGH
p = 0.13
gini_vertex_IGK
p = 0.22
gini_vertex_IGL
p = 0.16
gini_cluster_IGH
p = 0.0078
gini_cluster_IGK
p = 0.00058
gini_cluster_IGL
p = 6e−04
subtype
basal
classical
primitive
secretory

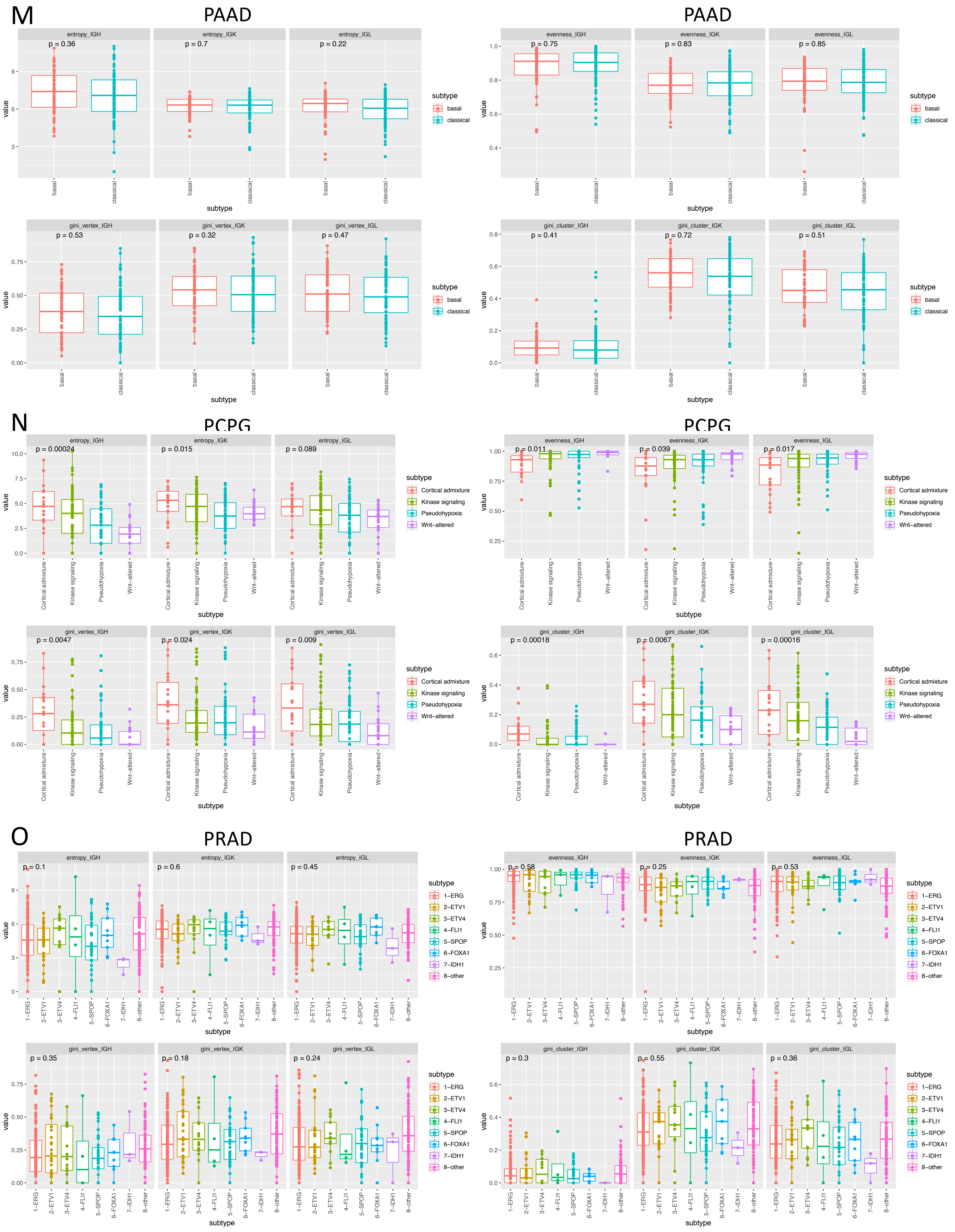
M
PAAD
entropy_IGH
entropy_IGK
entropy_IGL
p = 0.36
p = 0.7
p = 0.22
evenness_IGH
evenness_IGK
evenness_IGL
p = 0.75
p = 0.83
p = 0.85
gini_vertex_IGH
gini_vertex_IGK
gini_vertex_IGL
p = 0.53
p = 0.32
p = 0.47
gini_cluster_IGH
gini_cluster_IGK
gini_cluster_IGL
p = 0.41
p = 0.72
p = 0.51
subtype
basal
classical
value
N
PCPG
p = 0.00024
p = 0.015
p = 0.089
p = 0.011
p = 0.039
p = 0.017
p = 0.0047
p = 0.024
p = 0.009
p = 0.00018
p = 0.0067
p = 0.00016
Cortical admixture
Kinase signaling
Pseudohypoxia
Wnt−altered
O
PRAD
p = 0.1
p = 0.6
p = 0.45
p = 0.58
p = 0.25
p = 0.53
p = 0.35
p = 0.18
p = 0.24
p = 0.3
p = 0.55
p = 0.36
1−ERG
2−ETV1
3−ETV4
4−FLI1
5−SPOP
6−FOXA1
7−IDH1
8−other

P

SKCM SKCM

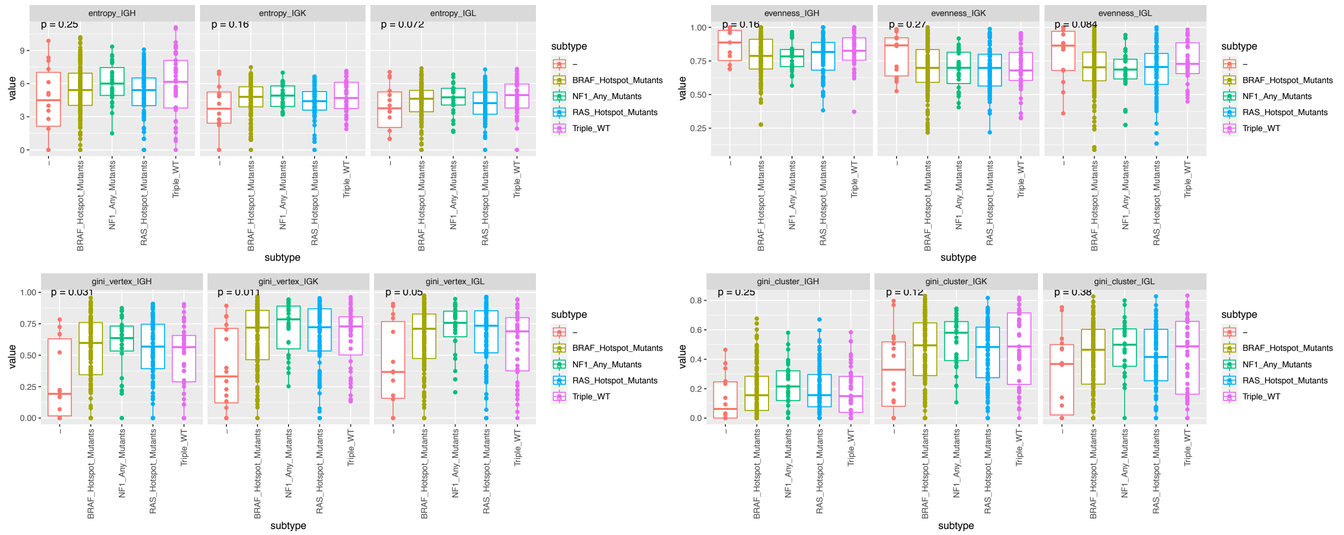

Q

THCA THCA

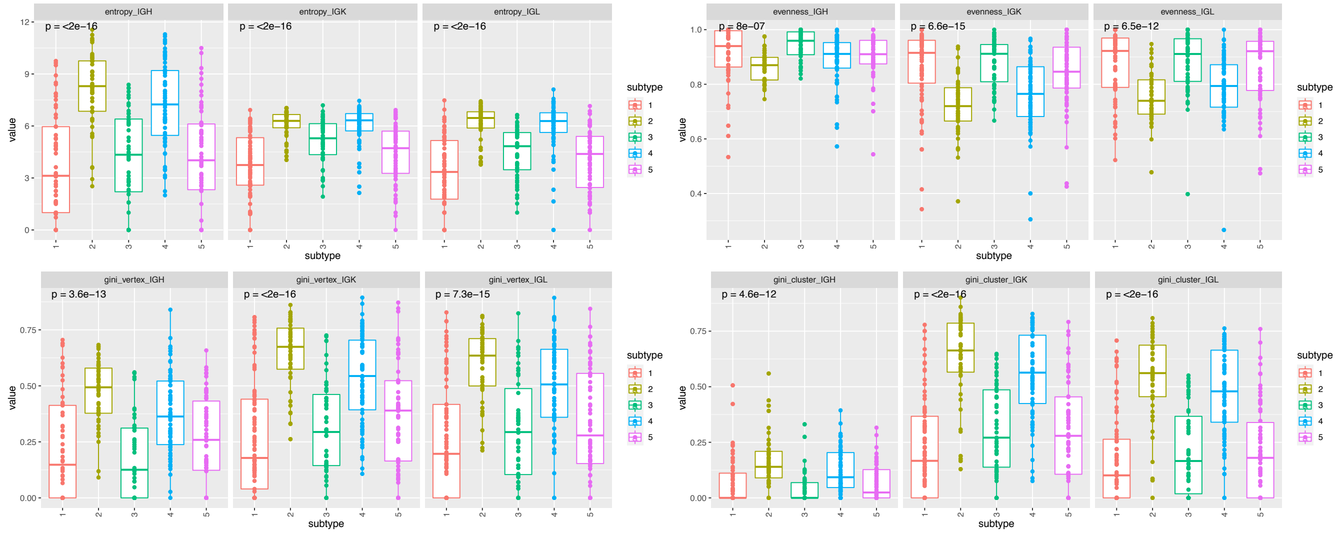

R

UCEC UCEC

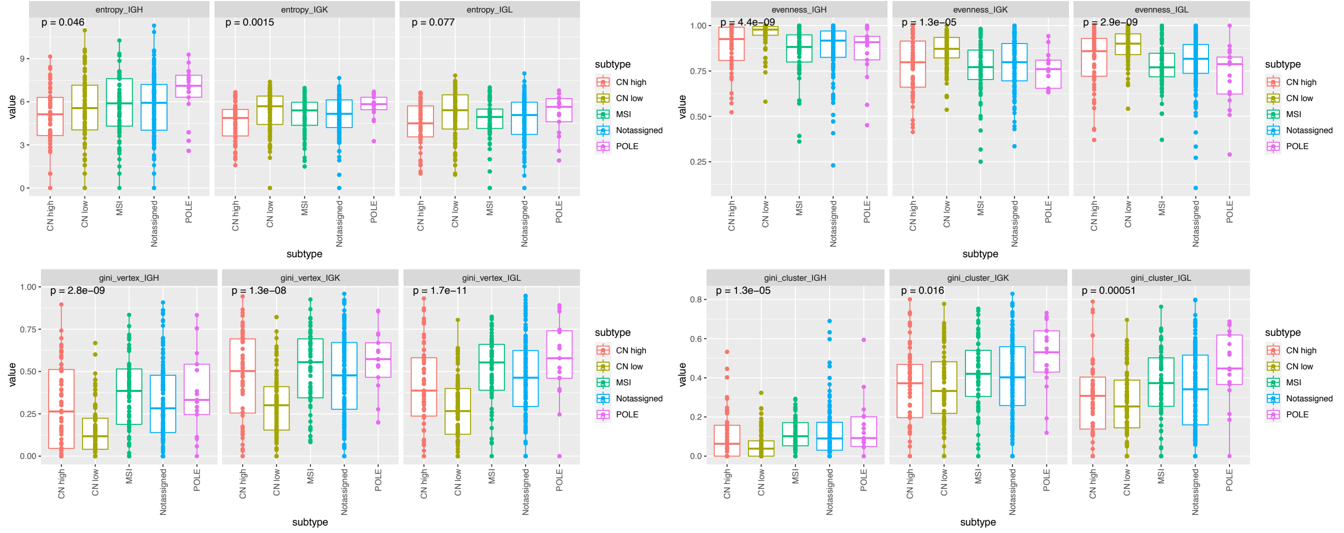

Supplementary Figure 4. Tumor subtype analysis of repertoire features. (a)-(r) Boxplots comparing the B cell repertoire features between tumor subtypes.

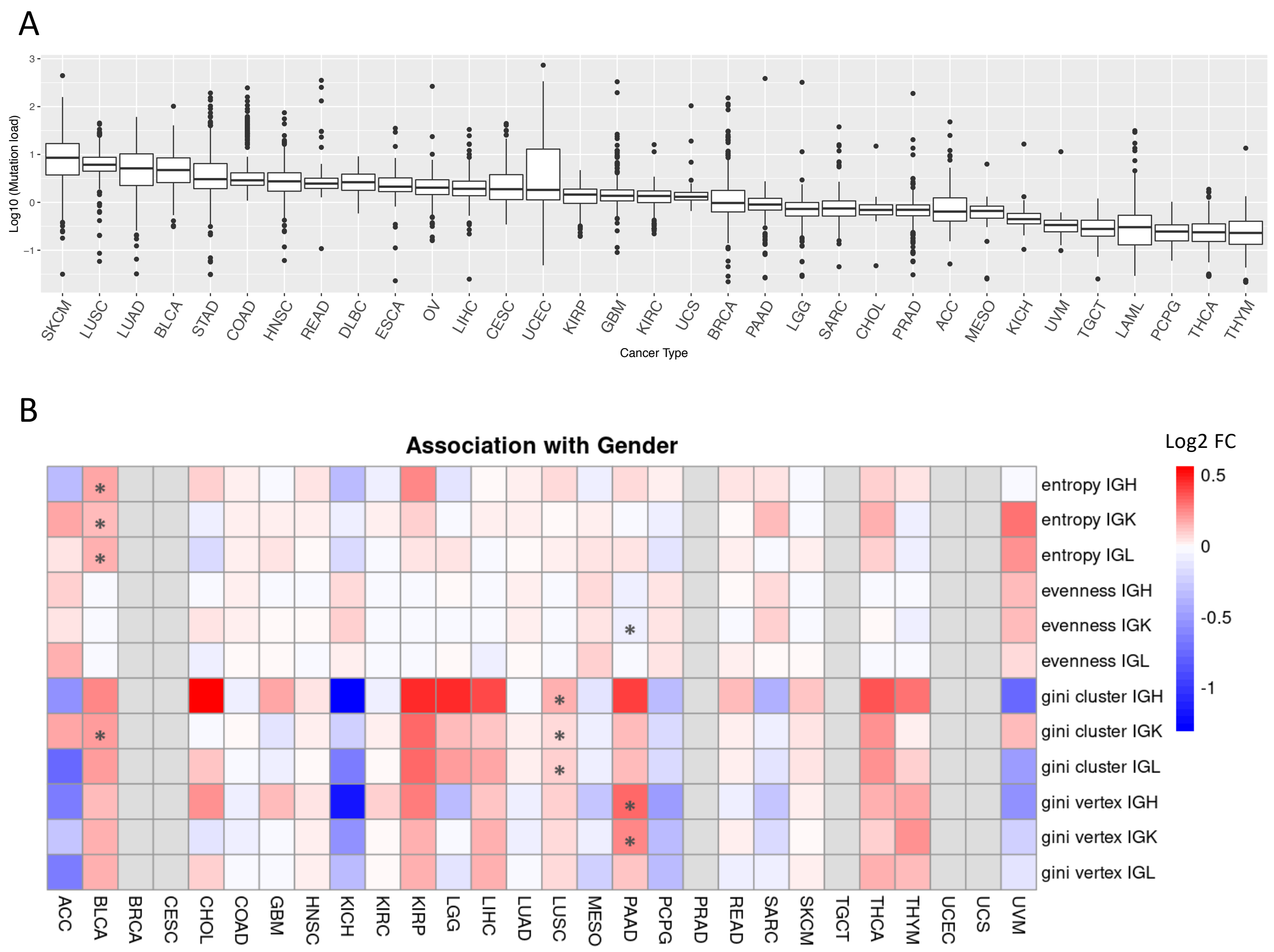

Supplementary Figure 5. Analysis of molecular and clinical features. (a) Boxplots of mutation load across tumor types. (b) Heatmap showing the log2 fold ratio between the mean value in females and the mean value in males. The Wilcoxon rank-sum test was used to calculate significance and significant comparisons with FDR < 0.05 are marked by an asterisk.

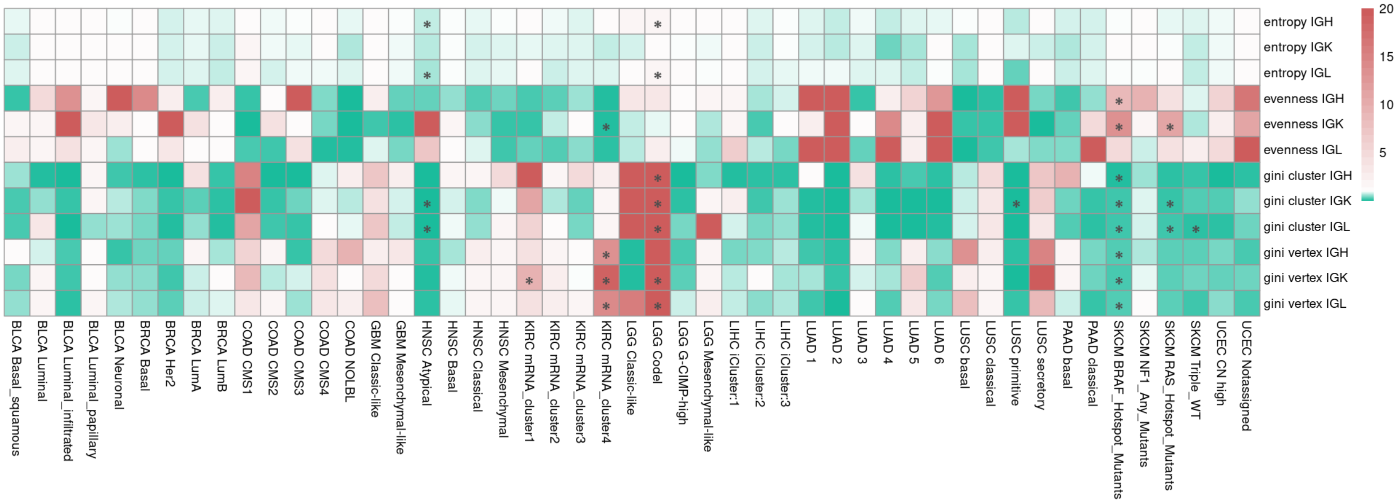

Supplementary Figure 6. Tumor subtype survival analysis. Heatmap shows the hazard ratio from a Cox proportional hazards model for each B cell repertoire feature. Columns are individual tumor subtypes. Red indicates a hazard ratio greater than 1 and green indicates a hazard ratio less than 1. Significant associations (FDR < 0.05) are marked by an asterisk.

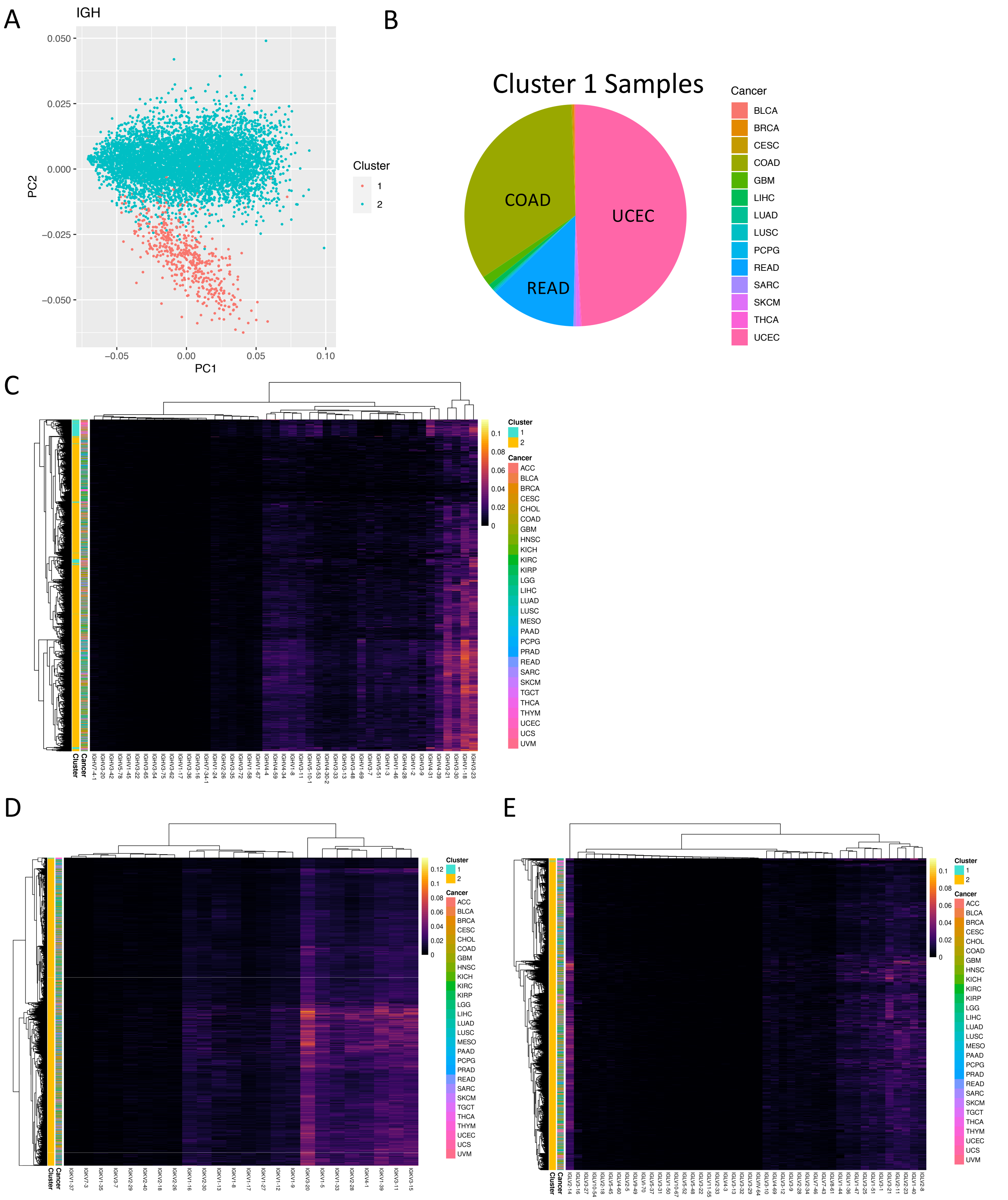

Supplementary Figure 7. V gene analysis. (a) PCA plot of IGH V gene usage. Samples are colored by the clusters identified by k-means clustering (k=2). (b) Pie chart showing the proportion of each tumor type in cluster 1. (c) Heatmap of IGH V genes associated with cluster 1. (d) Heatmap of IGK V genes associated with THYM samples. (e) Heatmap of IGL V genes associated with THYM samples.

Supplementary Table 1. Sequencing Summary

Tumor	Primary Tumor	Metastatic Tumors	Normal Solid	Total reads sequenced per sample	Ig Reads	Ig clones
				Median (min-max)	Median (min-max)	Median (min-max)
ACC	68	0	0	62615525 (26994182-93796872)	11 (0-9315)	8 (0-1025)
BLCA	407	0	19	59954267 (24027361-149204452)	1725 (0-288348)	319.5 (0-5261)
BRCA	1094	0	113	74886056 (24300451-187467995)	4908 (1-375918)	735 (1-15259)
CESC	303	0	3	66304315 (23950932-134684782)	4964.5 (9-230219)	657 (8-9865)
CHOL	35	0	9	55903461 (34208102-80520806)	636 (3-107785)	281 (2-11086)
COAD	456	0	41	51458173 (5271052-105993154)	4891 (8-120525)	795 (8-15477)
GBM	154	0	4	64703272.5 (44781738-120534895)	59.5 (1-19445)	41 (1-4285)
HNSC	520	0	44	69392642 (26296551-116430322)	4979 (3-327687)	647 (3-12053)
KICH	64	0	24	83461233 (46035744-106405236)	86 (0-48683)	50 (0-10999)
KIRC	532	0	72	78820886 (28668344-183724655)	1301 (1-430697)	360.5 (1-13868)
KIRP	288	0	32	67009890 (20901900-124057557)	186 (0-177328)	103 (0-20524)
LGG	438	0	0	72715489.5 (33127787-123178193)	4 (0-11434)	4 (0-1845)
LIHC	371	0	50	62932505 (25893240-153281376)	270 (0-85163)	104 (0-3682)
LUAD	515	0	59	57627892 (24146547-136132651)	17614 (15-315639)	1555 (12-16139)
LUSC	499	0	51	70169407.5 (21586543-199864020)	24190.5 (6-396978)	1976.5 (5-20364)
MESO	87	0	0	66384473 (33578727-90149604)	1728 (1-141587)	379 (1-8101)
PAAD	176	0	4	61316968.5 (23986132-108982216)	4413.5 (1-145633)	827 (1-7829)
PCPG	175	0	3	63274692.5 (37828408-118310186)	99 (1-47885)	64 (1-5045)
PRAD	497	0	52	67457442 (26403224-136690954)	394 (1-178640)	161 (1-10817)
READ	166	0	10	52522554 (19755698-117000539)	3741 (37-152400)	752 (21-10615)
SARC	258	0	2	63648060.5 (27256451-119791812)	130 (0-203957)	49.5 (0-11146)
SKCM	103	365	1	71509610 (8861327-134998914)	3849 (0-346317)	385 (0-12236)
TGCT	150	0	0	58327829.5 (27368204-107291085)	13119 (4-233460)	676 (3-5501)
THCA	337	0	38	79288399 (28396686-155154872)	722 (0-276438)	226.5 (0-17713)
THYM	120	0	2	63706807.5 (35195055-102344058)	1395 (0-301724)	327.5 (0-9985)

UCEC	545	0	35	34218459 (10058251-87154384)	1070.5 (1-261103)	278 (1-10145)
UCS	56	0	0	64390139.5 (43664951-75567157)	249 (2-123177)	63 (2-1789)
UVM	75	0	0	62680969 (31213794-89708332)	8 (0-157162)	7 (0-1061)
